# Pseudo-Jahn–Teller Effect in Natural Compounds and Its Possible Role in Straintronics I: Hypericin and Its Analogs

**DOI:** 10.3390/molecules29235624

**Published:** 2024-11-28

**Authors:** Dagmar Štellerová, Vladimír Lukeš, Martin Breza

**Affiliations:** Institute of Physical Chemistry and Chemical Physics STU, Radlinskeho 9, SK-81237 Bratislava, Slovakia; dagmar.stellerova@stuba.sk (D.Š.); vladimir.lukes@stuba.sk (V.L.)

**Keywords:** M06-2X hybrid functional, natural compounds, molecular symmetry, non-degenerate electronic states, excitation energy

## Abstract

The distortions and instability of high-symmetry configurations of polyatomic systems in nondegenerate states are usually ascribed to the pseudo-Jahn–Teller effect (PJTE). The geometries of hypericin, isohypericin, and fringelite D were optimized within various symmetry groups. Group-theoretical treatment and (TD-)DFT calculations were used to identify the corresponding electronic states during the symmetry descent. The symmetry descent paths (up to the stable structures without imaginary vibrations) were determined using the corresponding imaginary vibrations as their kernel subgroups starting from the highest possible symmetry group. The vibronic interaction between the ground and excited electronic states relates to an increasing energy difference of both states during the symmetry decrease. This criterion was used to identify possible PJTE. We have shown that the PJTE in these naturally occurring compounds could explain only the symmetry descent paths C_2v_ → C_2_ and C_2v_ → C_s_ in hypericin, and the D_2h_ → C_2v_, D_2h_ → C_2v_ → C_2_, and D_2h_ → C_2h_ ones in fringelite D. The electric dipole moments of hypericin and its analogs were determined prevailingly by the mutual orientations of the hydroxyl groups. The same held for the energies of frontier orbitals in these systems, but their changes during the symmetry descent were less significant.

## 1. Introduction

Straintronics represents a progressive field of modern condensed matter physics which investigates how physical effects in solids related to mechanical strain under the influence of external controlling fields in a layer of two-dimensional materials can change their electric, magnetic, optical, and other physical properties [1]. The aim of this investigation is to create prerequisites for the implementation of the new construction of sensors and electronics applicable in information technologies. Historically, the first materials, where these effects were studied, were of inorganic origin. These were mainly crystalline forms of germanium and silicon. Significant advances in this direction have been associated with two-dimensional materials such as graphene, hexagonal boron nitride, ZnO, ultrathin films of transition metal chalcogenides, semiconductor monolayers, and planar heterostructures on them [2]. The development of molecular electronics and instrumentation now allows for operation at distances less than 100 nanometers [3]. One of the biggest technical problems with working on single molecules is establishing reproducible electrical contact with only one molecule. Despite these difficulties, it is plausible to assume that straintronics will start to develop to the molecular level in the future. At this level, physical properties can be controlled by van der Waals interactions, acid–base processes, charge–transfer processes, or a combination of these. The use of this research can be expected, for example, in the design and control of molecular motors or robots [4]. The identification and investigation of organic molecules with a chemical structure suitable for potential applications in straintronics represents a significant challenge for theoretical chemistry. The desired structural motif may be based not only on synthetic molecules, but compounds of natural origin are also proving to be potentially interesting. The hypericin molecule, its derivatives, and corresponding tautomers turn out to be very interesting in this respect. This natural molecule is a derivative of anthraquinone and has antibacterial and antiviral effects [5]. It is a highly powerful biologically active compound. The existence of a large chromophore system in the molecule implies its photosensitivity. Hypericin was previously under research as an agent in photodynamic therapy.

**Figure 1:**
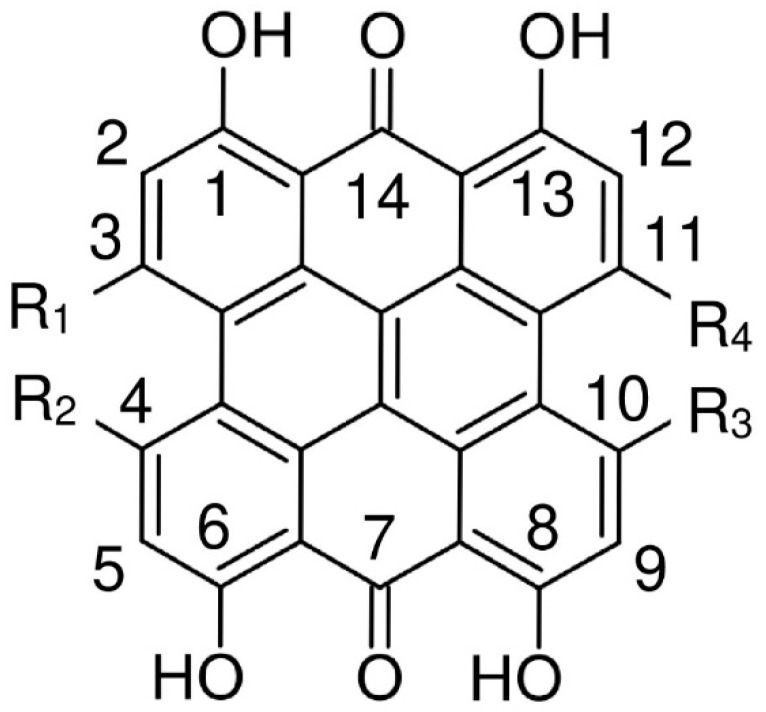
Atom notation of hypericin (R_1_ = R_2_ = OH, R_3_ = R_4_ = CH_3_), isohypericin (R_1_ = R_3_ = OH, R_2_ = R_4_ = CH_3_) and fringelite D (R_1_ = R_2_ = R_3_ = R_4_ = OH).

According to single-crystal X-ray measurements, the structure of hypericin 1,3,4,6,8,13-hexahydroxy-10,11-dimethylphenanthro[1,10,9,8-*opqra*]perylene-7,14-dione (**I**) [6] (see Figure 1) is non-planar. The ab initio Møller–Plesset method up to the second-order perturbation theory (MP2) [7] and numerous density functional theory (DFT) studies [8,9,10,11,12,13,14,15,16,17,18,19,20,21] have found stable ’propeller’ and ‘double butterfly’ conformations, with the latter being slightly lower in energy, which is consistent with X-ray structure data [6]. The nonplanar conformations are explained by steric repulsion between the hydroxyl groups in the 3, 4 positions and the methyl groups in the 10, 11 positions. The interconversion between them may occur at room temperature. Concerning possible hydrogen bonds, the most stable structure among 10 tautomers has quinone oxygens at the 7 and 14 positions. Many of these studies are focused on the hypericin spectral properties [10,11,12,15,17,18] with the aim of explaining its photodynamic activity.

To our best knowledge, no theoretical study dealing with the structure of isohypericin 1,3,4,6,8,10,13-hexahydroxy-4,11-dimethylphenanthro[1,10,9,8-*opqra*]perylene-7,14-dione (**II**) (see Figure 1) is available in the literature.

Their fully hydroxylated symmetric analogue fringelite D, 1,3,4,6,8,10,11,13-octahydroxyphenanthro[1,10,9,8-opqra]perylene-7,14-dione (**III**), (see Figure 1) was investigated only in a sole DFT study [12].

Although the distance between oxygen atoms O3-O4 as a measure of their mutual repulsion is evidently comparable with the remaining O-O distances in hypericins, their non-planarity is ascribed to the steric repulsion of hydroxyl groups in the 3 and 4 positions. An alternative explanation of this symmetry descent might be based on the pseudo-Jahn–Teller (PJT) effect (see later). According to Bersuker [22,23], “the pseudo-Jahn-Teller effect is the only source of instability and distortions of high-symmetry configurations of polyatomic systems in non-degenerate states”. The aim of our current study is to verify this hypothesis in the case of hypericin, isohypericin, and fringelite D. We used a group-theoretical treatment and quantum-chemical calculations at the DFT level for this purpose. Our methodology is of more general character to distinguish which distortions can be explained by the PJT effect and which cannot. Because these two-dimensional molecules represent suitable model compounds with potential future applications in straintronics, it is of interest to understand the extent to which the PJT effect or steric repulsion modulate molecular properties.

## 2. Theoretical Background

A potential energy surface (PES) of a molecule, an ion, a crystal, etc., describes its energy as a function of the coordinates of its atoms [24]. Geometry optimization is the process of finding PES stationary points (their gradients, i.e., the first partial derivatives of the energy with respect to all geometry parameters Q_i_ are equal to zero), such as its minima or saddle points. PES minima represent stable or quasistable species with energies E lower than those of its surrounding species (local minimum) or the lowest on the whole PES (absolute minimum). An n-th order saddle point has n negative eigenvalues of the Hessian matrix (∂^2^*E*/∂*Q*_i_∂*Q*_j_). Saddle points represent PES maxima along the directions of the reaction coordinates, and minima along all other directions. This means that saddle points represent transition states along the reaction coordinates.

A symmetry operation will be conserved during a nuclear displacement if and only if it leaves the displacement coordinate invariant [25,26]. A nondegenerate distortion coordinate Q_i_ described by a non-totally symmetric representation Γ_i_ within the symmetry group G_0_ causes the symmetry decrease from G_0_ to its kernel subgroup K(G_0_, Γ_i_), which consists of all symmetry elements with characters equal to +1. A degenerate representation describes a set of distortion coordinates and consists of several components that span a distortion space. An epikernel subgroup E(G_0_, Γ_i_) is conserved only in a part of the distortion space. Thus, epikernels are intermediate subgroups between the parent group G_0_ and the kernel subgroup, which is conserved in all distorted structures.

According to the Jahn–Teller (JT) theorem [27], any nonlinear configuration of atomic nuclei in a degenerate electronic state is unstable. Consequently, at least one stable nuclear configuration of lower symmetry must be obtained during a symmetry decrease where the degenerate electronic state is split. The PES of such systems can be described analytically using a perturbation theory treatment and its stationary points can be obtained [28]. The energy difference between the high-symmetry unstable and low-symmetry stable structures of the same compound is denoted as the Jahn–Teller stabilization energy *E*_JT_.

The classical JT effect is restricted to high-symmetry structures with degenerate electronic states, but a similar instability, known as the pseudo-Jahn–Teller (PJT) effect [23], may be observed in the case of sufficiently strong vibronic coupling between pseudodegenerate electronic states (usually ground and excited). JT active coordinate Q gives non-vanishing values of $<\Psi_{1}\left|\frac{\partial \hat{H}}{\partial Q}\right|\Psi_{2}>$ integrals between interacting electronic states *Ψ*_1_ and *Ψ*_2_, where $\hat{H}$ is Hamiltonian of the system under study. The PES of two interacting electronic states *Ψ*_1_ and *Ψ*_2_ of different space symmetries can be described in the simplest case using perturbation theory as

(1) $$E\left(Q\right)=\frac{1}{2}KQ^{2}\pm [\frac{{∆}^{2}}{4}+F^{2}Q^{2}{]}^{1/2}$$

where *E* is the energy of the electronic state, *Q* is the JT active distortion coordinate, Δ is the energy difference of both electronic states in the undistorted geometry (*Q* = 0), *K* is the primary force constant (without vibronic coupling), and *F* represents the vibronic coupling constant. The curvature ∂^2^*E*/∂*Q^2^* of the lower state for Q = 0 is negative (i.e., it corresponds to a PES saddle point) for

(2) $$∆<2\frac{F^{2}}{K}$$

Otherwise, the stable structure (the PES minimum) corresponds to *Q* = 0 and the high-symmetry structure is preserved (despite the lessened curvature of the lower-energy state). PJT interaction is restricted to electronic states of the same spin multiplicity, decreases with Δ and enhances the energy difference between the interacting electronic states.

The method of the epikernel principle [29] was originally elaborated to predict symmetry groups of stable JT structures and has been extended to PJT systems as well [30]. It is based on the representation Γ_JT_ of the JT active coordinate Q that gives non-vanishing values of $<\Psi_{1}\left|\frac{\partial \hat{H}}{\partial Q}\right|\Psi_{2}>$ integrals between interacting electronic states *Ψ*_1_ and *Ψ*_2_ described by representations Γ_1_ and Γ_2_, respectively. For the totally symmetric Hamiltonian $\hat{H}$, it implies that Γ_JT_ is contained in the direct product of representations of both interacting electronic states

Γ_JT_ є Γ_1_ ⊗ Γ_2_ (3)

Analogously, for the classical JT effect with a degenerate electronic state *Ψ*_1_ = *Ψ*_2_ and Γ_1_ = Γ_2_ = Γ, an even more strict condition is valid, and the Γ_JT_ must be contained in its symmetric direct product.

Γ_JT_ є [Γ ⊗ Γ]^+^ (4)

According to the epikernel principle, the JT stable structures correspond to the kernel K(G_0_, Γ_JT_) or (preferably) the epikernel E(G_0_, Γ_JT_) subgroups of the parent group G_0_.

Classical JT vibronic interactions can be identified according to a single criterion—the degenerate electronic state. On the other hand, the identification of PJT vibronic interactions is a more complicated task that consists of several steps. For simplicity, we will restrict our analysis to interactions between the ground state *Ψ*_1_ and the excited state(s) *Ψ*_2_. At first, the geometry of the studied system is optimized within its highest symmetry group, the symmetries of imaginary vibrations are identified by vibrational analysis, and subsequently, the symmetries and energies of its excited states are evaluated. For known symmetries of an imaginary vibration (tentatively identified with a JT active coordinate) and of the ground state, we can determine the excited-state symmetry according to Equation (3). In the next step(s), we repeat the geometry optimization (the energy of the system in the parent group must be higher than in its subgroups). Then, we determine the symmetries of imaginary vibrations, the energies, and symmetries of the excited states of the kernel and epikernel subgroups of the parent high-symmetric group. Subsequently, we identify the corresponding excited states in the parent group and its subgroups according to the group—subgroup relations (see Tables S1–S3 in Supplementary Information) [31], similar oscillator strengths, and similar molecular orbital compositions. This step might be problematic because—based on the perturbation principle—a high similarity between the corresponding excited states is supposed. The PJT vibronic interaction causes the excitation energy of the corresponding excited state in the PJT subgroup to be higher than in the parent group (compare Equation (1)). Otherwise, the investigated symmetry descent cannot be a consequence of the PJT vibronic interaction, and other explanations must be searched for.

## 3. Results and Discussion

All the molecules under study are neutral closed-shell systems in the ground singlet spin state. Thus, their ground spin states are described by total symmetric representations independent of the symmetry group. It implies that the representation of the PJT interacting excited state and of the JT active coordinate are equal (see Equation (3)).

We started our study with geometries of the highest possible symmetry with quinone oxygens at the 7 and 14 positions (see Figure 1). According to [17], this tautomer corresponds to the most stable structure of hypericin.

Although we performed time dependent (TD)-DFT calculations for 50 excited states, only the lowest 10 excited states are presented. Our analysis is restricted to the lowest excited state of every symmetry, as the vibronic interaction with this state is supposed to be stronger than with the higher ones. The notation n^m^Γ(G) is used for the n-th state of Γ representation with spin multiplicity m within the G symmetry group.

Our results are discussed in several steps. At first, the imaginary vibrations and corresponding kernel subgroups of the parent structures were identified using the tables in the main text. Subsequently, we change to the tables in Supplementary Information, where we found the lowest excited state of a suitable symmetry (identical with the imaginary vibration) in the parent group, and its counterpart in the kernel subgroup was identified on the basis of group–subgroup relations and the similarity of their electron transitions and oscillator strengths. Finally, we compared the excitation energies of both electron states. The whole procedure was repeated for each step of every symmetry descent path.

### 3.1. Hypericin (I)

**Figure 2:**
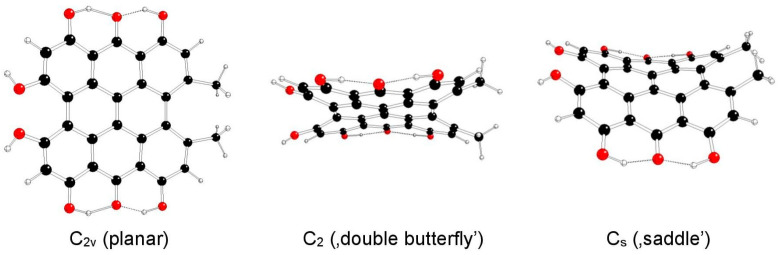
Optimized structures of hypericin Ia model systems (C—black, O—red, H—white).

**Figure 3:**
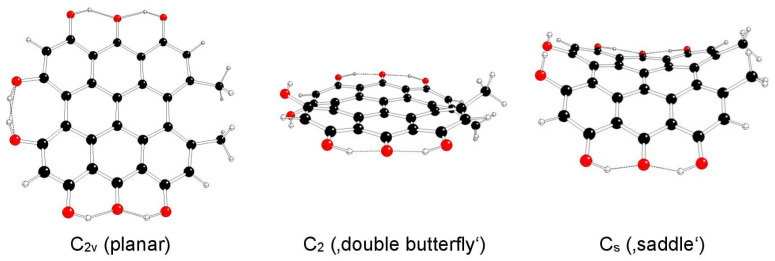
Optimized structures of hypericin Ib model systems (C—black, O—red, H—white).

**Figure 4:**
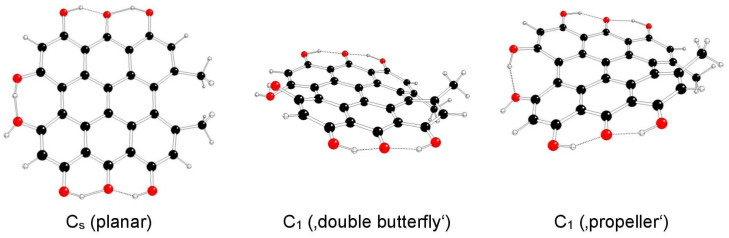
Optimized structures of hypericin Ic model systems (C—black, O—red, H—white).

The highest possible symmetry group of hypericin (I) is C_2v_. Here, both its hydroxyl groups at positions 3 and 4 are either in *anti-* or *syn-* mutual orientations and denoted as models Ia and Ib, respectively (Figure 2 and Figure 3). The third possible mutual orientation (one in *syn*- and the other in mutual *anti*-orientations) is denoted as Ic, with the highest possible symmetry group C_s_ (Figure 4). The non-symmetric structure (C_1_) cannot be found for the Ia and Ib model systems, but it corresponds to the Ic model system only. This assignment is implied by the highest gradients at the H atoms of the hydroxyl groups at the 3 and 4 positions of unstable Ia and Ib structures in the C_s_ group.

Table 1 illustrates the energy decrease of hypericin model systems Ia, Ib, and Ic with their decreasing symmetry as implied by imaginary vibrations (corresponding kernel subgroups). The increasing distances between hydroxyl O atoms in the 3 and 4 positions d_OO_ illustrate their decreasing mutual repulsion with symmetry decrease, which coincided with possible JT stabilization energies. The most stable Ic model systems (especially the C_1_ ‘propeller’) were stabilized by an additional hydrogen bond (compare Figure 4). The mutual orientations of hydroxyl groups in the 3 and 4 positions affected the electric dipole moments and the frontier orbital (HOMO = the Highest Occupied Molecular Orbital, LUMO = the Lowest Unoccupied Molecular Orbital) energies (Table S4 in Supplementary Information). The C_s_ symmetry structures had the highest dipole moments. The orbital energies decreased in the order Ia > Ic > Ib, and their dependence on symmetry was less significant.

Individual symmetry descent paths are summarized in Table 2. The possible PJT origin of the observed symmetry descent paths can be checked with the help of Table S5 in Supplementary Information as follows.

The symmetry descent no. 1 can be ascribed to the vibronic interaction of X^1^A_1_(C_2v_) ground state with the 1^1^A_2_(C_2v_) excited state, which corresponds to the 4^1^A(C_2_) excited state with increased excitation energy in both the Ia and Ib systems.

The two-step symmetry decrease no. 2 meets the problem with its excited state identification. Nevertheless, using an elimination method, we can conclude that the HOMO-7 → LUMO electron transition generating the 1^1^B_1_(C_2v_) excited state corresponded to the HOMO-8 → LUMO electron transition generating the 4^1^A’(C_s_) excited state with the higher excitation energy, and so the PJT effect was possible. The structure obtained in this way was unstable, but the supposed vibronic interaction of the X^1^A’(C_s_) ground state with the 1^1^A”(C_s_) led to the 2^1^A(C_1_-‘propeller’) or 3^1^A(C_1_-‘double butterfly’) excited state with lower excitation energies. Therefore, this second step cannot be explained by the PJT effect.

In the first step of the symmetry descent no. 3, the X^1^A_1_(C_2v_) ground state interacted with the 1^1^B_2_(C_2v_) excited state, which corresponded to the 1^1^A”(C_s_) excited state with higher excitation energy, and the PJT vibronic interaction was possible. However, this structure was unstable, and this state corresponded to the 2^1^A(C_1_) state, which had lower excitation energy in both ‘propeller’ and ‘double butterfly’ structures. Thus, this step cannot be explained by the PJT effect.

Within the symmetry descent no. 4, the X^1^A’(C_s_) ground state could interact with the 11A”(C_s_) excited state. It was problematic to find its corresponding excited state in the C_1_ kernel subgroup, but it was surely lower than the 8^1^A(C_1_) state, and therefore its excitation energy was lower. Consequently, this symmetry descent cannot be explained by the PJT effect.

### 3.2. Isohypericin (II)

**Figure 5:**
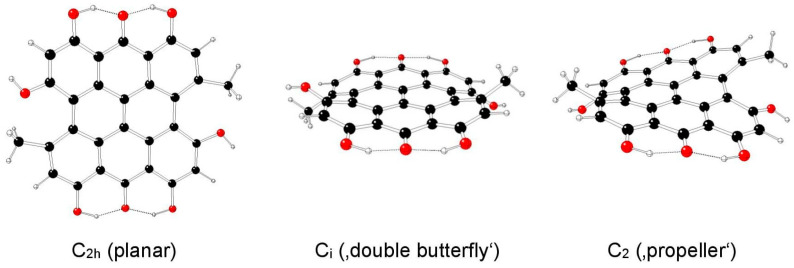
Optimized structures of isohypericin IIa model systems (C—black, O—red, H—white).

**Figure 6:**
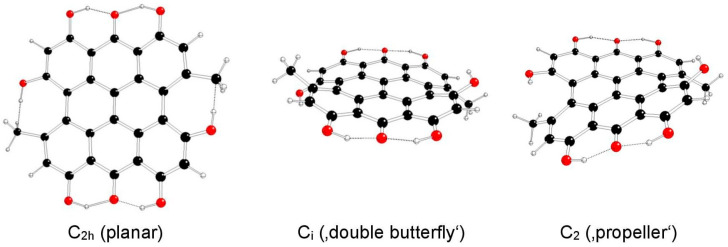
Optimized structures of isohypericin IIb model systems (C—black, O—red, H—white).

**Figure 7:**
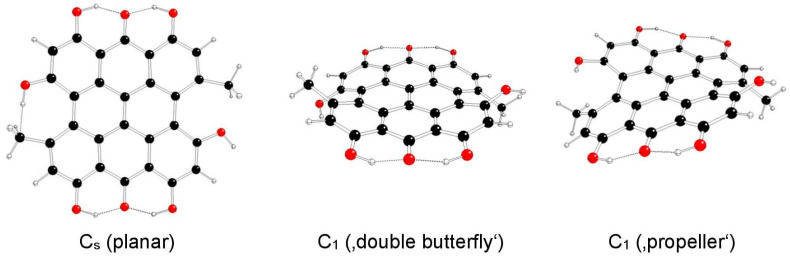
Optimized structures of hypericin IIc model systems (C—black, O—red, H—white).

The highest possible symmetry group of isohypericin (II) is C_2h_ if both of its hydroxyl groups at the 3 and 10 positions are related to methyl groups at the 4 and 11 positions either in *anti-* or *syn-* orientations and are denoted as models IIa and IIb, respectively (Figure 5 and Figure 6). The third possible orientation (one in *syn-* and the other in *anti*-orientations to methyl groups) is denoted as IIc, with the highest possible symmetry group C_s_ (Figure 7).

Table 3 illustrates the energy decrease of isohypericin model systems IIa, IIb, and IIc, with their decreasing symmetry as implied by imaginary vibrations (corresponding kernel subgroups). The increasing distances between hydroxyl O atoms in the 3 (11) and methyl C atoms in the 4 (10) positions d_OC_ illustrate their decreasing mutual repulsion with symmetry decrease, which coincided with possible JT stabilization energies. The most stable were ‘propeller’ structures, especially in the IIb model systems.

The centrosymmetric C_2h_ and C_i_ structures had no dipole moments (Table S6 in Supplementary Information). The mutual orientation of hydroxyl groups in the 3 and 10 positions affected the dipole moments (the highest dipole had the IIc structure of the symmetry C_s_) and the frontier orbital energies (HOMO, LUMO) (Table S6 in Supplementary Information). The orbital energies decreased in the order IIa > IIc > IIb, and their dependence on symmetry was less significant.

The possible symmetry descent paths of isohypericin are presented in Table 3 and Table 4. The possible PJT origin of the observed symmetry descent paths can be checked with the help of Table S7 in Supplementary Information as follows.

The symmetry descent path no. 5 might be explained by vibronic interaction of the ground electronic state X^1^A_g_(C_2h_) with the excited state 1^1^B_g_(C_2h_) (the HOMO-8 → LUMO electron transition in **IIa** and the HOMO-6 → LUMO one in **IIb**), which corresponded to the 4^1^A_g_(C_i_) excited state with lower excitation energy. This excluded the PJT effect as the reason for this symmetry descent. Using an elimination method for IIb, we can show that the HOMO-6 → LUMO electron transition (1^1^B_g_(C_2h_) excited electronic state) corresponded to the HOMO-8 → LUMO electron transition (the 4^1^A_g_(C_i_) excited state) with lower excitation energy, and so this symmetry descent cannot be of PJT origin.

The symmetry descent path no. 6 for both **IIa** and **IIb** systems might have originated in vibronic interaction of the ground state X^1^A_g_(C_2h_) with the excited state 1^1^B_u_(C_2h_), which corresponded to the 4^1^A(C_2_) excited state with lower excitation energy. Thus, the PJT origin of this symmetry descent was excluded.

The symmetry descent no. 7 (model system **IIc**) met the problem of excited state identification. The most probable assignment of the 1^1^A’(C_s_) excited state generated by the HOMO-7 → LUMO electron transition is the 7^1^A(C_1_) electronic state generated mainly by the HOMO-8 → LUMO electron transition in both ‘propeller’ and ‘double butterfly’ forms of the C_1_ kernel subgroup, which had lower excitation energy. Thus, the PJT origin of this symmetry descent can be rejected.

### 3.3. Fringelite D (III)

**Figure 8:**
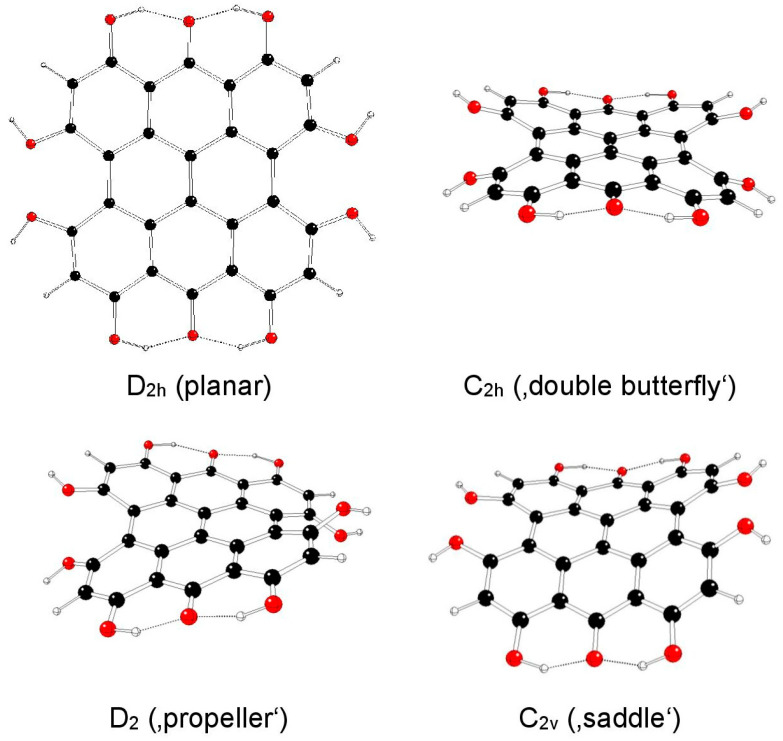
Optimized structures of fringelite D IIIa model systems (C—black, O—red, H—white).

**Figure 9:**
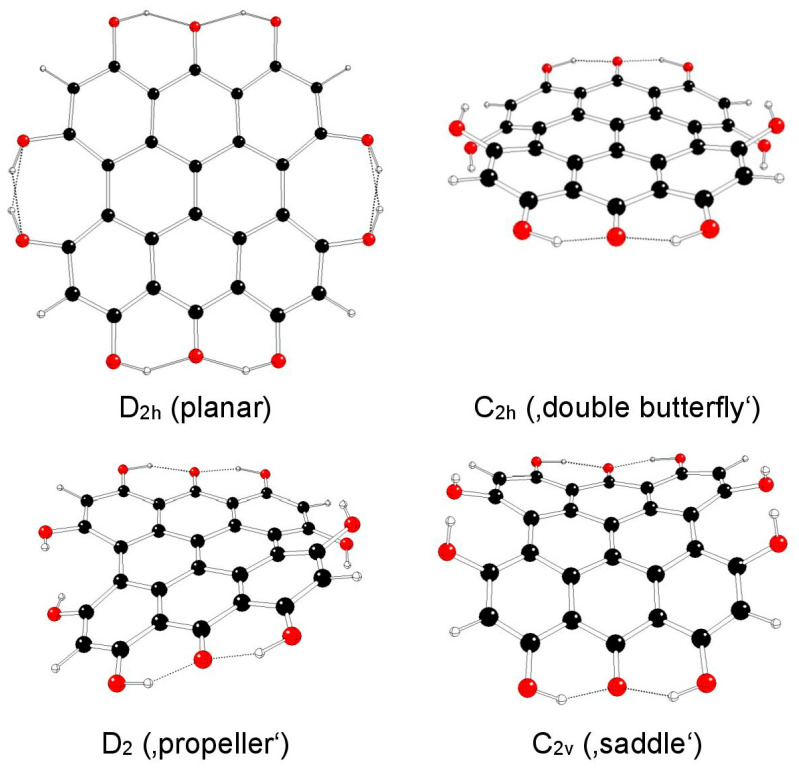
Optimized structures of fringelite D IIIb model systems (C—black, O—red, H—white).

**Figure 10:**
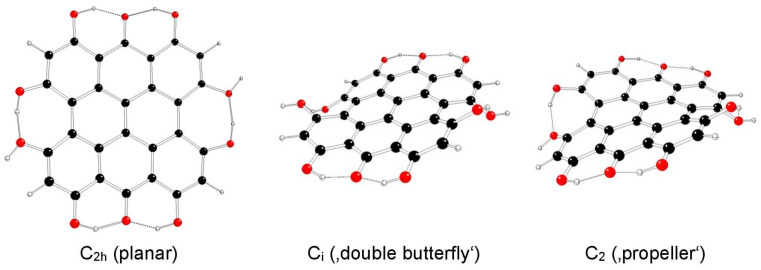
Optimized structures of fringelite D IIIc model systems (C—black, O—red, H—white).

**Figure 11:**
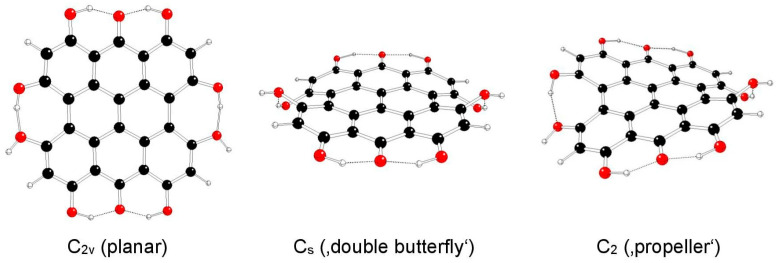
Optimized structures of fringelite D IIId model systems (C—black, O—red, H—white).

The highest possible symmetry group of fringelite D (III) is D_2h_ if both its hydroxyl groups at the 3 and 4 as well as in the 10 and 11 positions are either in *anti*- or *syn*- mutual orientations. They are denoted as models IIIa and IIIb, respectively (Figure 8 and Figure 9). Other possible mutual orientations are denoted as IIIc (*syn*-orientations at the 3 and 10 positions, *anti*-orientations at the 4 and 11 positions), with the highest possible symmetry group C_2h_, and IIId (*syn*-orientations at the 3 and 11 positions, *anti*-orientations at the 4 and 10 positions), with the highest possible symmetry group C_2v_ (Figure 10 and Figure 11). The structures of C_s_ and C_2_ symmetry groups could not be found for IIIa and IIIb model systems, and so they corresponded to those of IIIc and IIId systems. This assignment was supported by the highest gradients at H atoms of hydroxyl groups at the 3, 4, 10, and 11 positions of the unstable IIIa and IIIb structures in the C_2v_ group.

Table 5 illustrates the energy decrease of fringelite D model systems IIIa, IIIb, IIIc, and IIId with their decreasing symmetry as implied by imaginary vibrations (corresponding kernel subgroups). The increasing distances between hydroxyl O atoms in the 3 and 4 (or 10 and 11) positions d_OO_ illustrate their decreasing mutual repulsion with symmetry decrease, which coincided with possible JT stabilization energies. The increased stability of IIIc and IIId systems was caused by additional hydrogen bonds of hydroxyl groups in the 3 and 4 as well as in the 10 and 11 positions. The most stable were ‘propeller’ structures, especially in IIIc model systems.

The D_2h_, D_2_, C_2h_, and C_i_ structures had no dipole moments (Table S8 in Supplementary Information). The mutual orientations of hydroxyl groups in the 3, 4, 10, and 11 positions affected the dipole moments (the highest dipole had the IIId structure of C_2v_ symmetry) and the frontier orbital energies (Table S8 in Supplementary Information). The orbital energies decreased in the order IIIa > IIIc ~ IIId > IIIb, and their dependence on symmetry was less significant.

Individual symmetry descent paths are summarized in Table 6. The possible PJT origin of the observed symmetry descent paths can be checked with the help of Table S9 in Supplementary Information as follows.

The lowest excited 1^1^B_2g_(D_2h_) state (no. 37) of IIIa and IIIb compounds which was capable of vibronic interaction with the X^1^A_g_(D_2h_) ground electronic state within the descent path no. 8 was too high (the HOMO-22 → LUMO+8 electron transition was over 6.1 eV) to be PJT active. It was confirmed by the lower excitation energy of its 8^1^A_g_(C_2h_) counterpart for the IIIa structure. We have not found its counterpart for the IIIb one.

The symmetry descent path no. 8 can be explained by the vibronic interaction of the X^1^A_1g_(D_2h_) ground state with the 1^1^A_u_(D_2h_) excited state in the IIIb system only (the electron transition HOMO-9 → LUMO) because its corresponding 2^1^A(D_2_) state in the IIIb system had lower excitation energy. It implies that there was no PJT effect.

The symmetry descent path no. 10a consisted of two steps. In IIIa, the X^1^A_g_(D_2h)_ ground state might have, at first, interacted with the 1^1^B_3u_(D_2h_) excited state, which corresponded to the 10^1^A_1_(C_2v_) state with higher excitation energy (the HOMO-9 → LUMO + 2 electron transition with the excitation energy over 6.5 eV). In the next step, the 1^1^B_2_(C_2v_) excited state (the HOMO-2 → LUMO electron transition) corresponded to 1^1^A’(C_s_) state for IIIc with lower excitation energy, which means no PJT interaction. In IIIb, the excited state 1^1^B_3u_(D_2h_) (the HOMO-1 → LUMO electron transition) corresponded to the 1^1^A_1_(C_2v_) state with higher excitation energy, which allowed the PJT interaction. In the second step, the 1^1^B_2_(C_2v_) excited state (the HOMO → LUMO electron transition) corresponded to the 1^1^A”(C_s_) excited state with lower excitation energy, which excluded the PJT interaction.

The first step of the 10b symmetry descent path for the IIIa and IIIb systems was the same as in 10a. In the second step, the 1^1^A_2_(C_2v_) excited state (the HOMO → LUMO + 1 electron transition) corresponded to the 3^1^B(C_2_) one with higher excitation energies of both IIIc and IIId systems, allowing the PJT interaction.

The IIIb system underwent the symmetry descent no. 11, but it met the problem with the excited state identification. The most probable assignment of the 1^1^B_3g_(D_2h_) excited state generated by the HOMO-7 → LUMO electron transition corresponded to the 3^1^B_g_(C_2h_) electronic state generated by the HOMO-8 → LUMO electron transition with higher excitation energy, which allowed the PJT interaction.

The symmetry descent paths 12a and 12b of IIIb consisted of two steps. In the first step, the 1^1^B_u_(D_2h_) excited state (the HOMO- 9 → LUMO+2 electron transition, excitation state no. 46 with more than 6.3 eV excitation energy) corresponded to the 10^1^A_1_(C_2v_) state. This higher excitation energy allowed the PJT interaction. In the second step of the 12a path, the 1^1^B_2_(C_2v_) excited state (the HOMO → LUMO excitation) corresponded to the 1^1^A”(C_s_) state of the IIId system. This lower excitation energy excluded the PJT interaction. In the second step of the 12b path, the 1^1^A_2_(C_2v_) state of IIIb (the HOMO → LUMO+1 electron excitation) corresponded to the 3^1^B(C_2_) state of IIId or to the 3^1^A(C_2_) state of IIIc with higher excitation energies. Here, the PJT effect was possible. The 3^1^B(C_2_) state of IIId did not satisfy the group–subgroup relations (see Table S3 in Supplementary Information).

In the symmetry descent path no. 13, the 1^1^B_1g_(D_2h_) state of the IIIb system (the HOMO-4 → LUMO and HOMO → LUMO+1 electron transitions) could not correspond to the 2^1^A_u_(C_2h_) state because of incorrect group–subgroup relations. However, the 2^1^B_1g_(D_2h_) state obtained by similar electron transitions corresponding to the 2^1^B_g_(C_2h_) state of the same IIIb system met all conditions for PJT interactions.

The symmetry descent paths 14a and 14b consisted of two steps. At first, the 1^1^B_3u_(D_2h_) excited state (the HOMO-1 → LUMO electron transition) coincided with the 1^1^B_1_(C_2v_) state with higher excitation energy in agreement with the PJT interactions. The path 14a continued with the 1^1^B_2_(C_2v_) electronic state of IIIb (the HOMO →LUMO electron transition) to the 1^1^A”(C_s_) state in the IIId system with lower excitation energy that excluded the PJT interaction. The path 14b continued with the 1^1^A_2_(C_2v_) excited state of the IIIb system (the HOMO →LUMO+1 electron transition) to the corresponding 3^1^A(C_2_) state of the IIIc system with higher excitation energy in agreement with the PJT interaction. The 3^1^B(C_2_) state of the IIId system did not fulfill the group–subgroup relations (see Table S3 in Supplementary Information).

In the symmetry descent path no. 15, the interaction of the X^1^A_g_(C_2h_) ground state with the 1^1^B_g_(C_2h_) excited state of the IIIc system (the HOMO-8 → LUMO electron transition) would lead to its 3^1^A_g_(C_i_) state with lower excitation energy, which excluded the PJT interaction.

The symmetry descent path no. 16 might have been due to the 1^1^A_u_(C_2h_) excited state. This transition came from HOMO-9 → LUMO electron excitation. It corresponded to the 4^1^A(C_2_) state of the IIIc system with lower excitation energy, which was in contradiction to the PJT effect.

The symmetry descent no. 17 cannot be ascribed to the PJT effect. It corresponded to the hypothetical interaction of the X^1^A_1_(C_2v_) ground state, with the very high excited state 1^1^B_1_(C_2v_) (state no. 37) corresponding to the 16^1^A’(C_s_) state of the IIId system with a lower excitation energy, excluding the vibronic interaction.

The same holds for the symmetry descent no. 18, where the 1^1^A_2_(C_2v_) state (the HOMO-7 → LUMO electron transition) corresponded to the 3^1^A(C_2_) state of the IIId system with lower excitation energy and thus without the PJT effect.

## 4. Method

The geometries of the neutral hypericin (**I**), isohypericin (**II**), and fringelite D (**III**) in the ground spin state were optimized within various symmetry groups using the M06-2X hybrid functional [32] and standard cc-pVDZ basis sets for all atoms taken from the Gaussian library [33]. The M06-2X functional is a highly non-local functional with doubled non-local exchange and with dispersion correction. It gives the best performance for unimolecular and association barrier height calculations in the DBH76 database, and its valence and Rydberg transitions perform as the third best among the compared DFT functionals in the VRES41 database [32].

The optimized geometries were checked for imaginary vibrations by vibrational analysis. Time-dependent DFT (TD-DFT) treatment [34,35] for up to 50 vertical electronic states was used to obtain excitation energies and excited states. Gaussian16 software (Revision B.01) [33] was used for all quantum-chemical calculations. The MOLDRAW software (https://moldraw.software.informer.com/, accessed on 9 September 2019) [36] was used for geometry manipulation and visualization purposes.

## 5. Conclusions

The group-theoretical treatment and quantum-chemical calculations were used to identify the corresponding electronic states of natural compounds hypericin (**I**), isohypericin (**II**), and fringelite D (**III**) in the structures of various symmetry groups. The symmetry descent paths (up to the stable structures without any imaginary vibrations) were determined using the corresponding imaginary vibrations as their kernel subgroups starting from the highest possible symmetry group for the given orientations of the hydroxyl groups at the 3, 4, 10, and/or 11 positions. Because the vibronic interaction between the ground and excited electronic states was connected with increasing excitation energy (the energy difference of both states) during the symmetry decrease (see above), this criterion was used for the identification of the possible PJT effect (this criterion was not mandatory if the symmetry descent was due to the mutual repulsion of hydroxyl and/or methyl groups). Our results indicated that the PJT effect explained only the symmetry descent paths C_2v_ → C_2_ and C_2v_ → C_s_ in hypericin, the D_2h_ → C_2v_, D_2h_ → C_2v_ → C_2_, and the D_2h_ → C_2h_ ones in fringelite D. Moreover, this criterion may have been satisfied accidentally in some cases. No symmetry descent path could be ascribed to the PJT effect in isohypericin, in the **Ic** model systems of hypericin, and in the **IIIc** and **IIId** model systems of fringelite D. Our results are in contradiction with Bersuker’s conception of the “high-symmetry configuration”, where “all its Coloumb and steric forces are equilibrated and if after that the symmetry of the system breaks, this spontaneous symmetry breaking is of pseudo-Jahn-Teller origin” [37].

Our results showed that the dipole moments of hypericin and its analogs were determined prevailingly by the mutual orientations of the hydroxyl groups. The same held for the energies of frontier orbitals in these systems, and their changes during the symmetry descent were less significant independent of the origin of the corresponding symmetry descent paths. These findings might be important for the future development of straintronics materials.

In this study, we proposed the methodology to distinguish the structural consequences of the PJT effect from those caused by mutual repulsion of relevant functional groups. We propose that its applications to less symmetric, naturally occurring molecules might be interesting from the point of view of the PJT effect. Further experimental and theoretical studies in this field are desirable.

## Figures and Tables

**Table 1:** Hypericin model systems of the symmetry groups G and the corresponding electronic ground states Γ_gr_, with DFT energies E_DFT_, JT energies E_JT_ related to the parent group G_0_, distances between hydroxyl O atoms at the 3 and 4 positions d_OO_, and symmetries Γ_im_ and wavenumbers ν_im_ of the corresponding imaginary frequencies and resulting kernel subgroups K (G_0_, Γ_im_).

Model	G	Γ_gr_	E_DFT_ (Hartree)	E_JT_ (eV)	d_OO_ (Å)	Γ_im_	ν_im_ (cm^−1^)	K(G_0_, Γ_im_)
G_0_ = C_2v_(**Ia**)								
**Ia**	C_2v_^(a)^	X^1^A_1_	−1754.24175	0.000	2.253	a_2_	−169, −132, −26	C_2_
						b_1_	−51, −35	C_s_
**Ia**	C_2_^(b)^	X^1^A	−1754.31051	1.871	2.515	-	-	-
**Ia**	C_s_^(c)^	X^1^A’	−1754.26014	0.500	2.363	a”	−114, −86	C_1_
**Ic**	C_1_^(d)^	X^1^A	−1754.31780	2.069	2.547	-	-	-
**Ic**	C_1_^(b)^	X^1^A	−1754.31525	2.000	2.534	-	-	-
G_0_ = C_2v_(**Ib**)								
**Ib**	C_2v_^(a)^	X^1^A_1_	−1754.19146	0.00	2.931	a_2_	−1469, −174, −147, −35	C_2_
						b_2_	−660	C_s_
						b_1_	−653, −57, −53	C_s_
**Ib**	C_2_^(b)^	X^1^A	−1754.31497	3.361	2.609	-	-	-
**Ib**	C_s_^(c)^	X^1^A’	−1754.24669	1.503	2.566	a”	−530, −112, −106	C_1_
**Ic**	C_1_^(b)^	X^1^A	−1754.31525	3.368	2.534	-	-	-
**Ic**	C_1_^(d)^	X^1^A	−1754.31780	3.438	2.547	-	-	-
G_0_ = C_s_(**Ic**)								
**Ic**	C_s_^(a)^	X^1^A’	−1754.25393	0.000	2.396	a”	−169, −123, −52, −36, −26	C_1_
**Ic**	C_1_^(d)^	X^1^A	−1754.31780	1.738	2.547	-	-	-
**Ic**	C_1_^(b)^	X^1^A	−1754.31525	1.669	2.534	-	-	-

Remarks: ^(a)^ planar conformation. ^(b)^ ‘double butterfly’ conformation. ^(c)^ ‘saddle’ conformation. ^(d)^ ‘propeller’ conformation.

**Table 2:** Symmetry descent paths of hypericin model systems. Possible PJT descent paths are in bold.

No.	Systems	Descent Path
1	**Ia**, **Ib**	**C_2v_** $\overset{a_{2}}{\to }$ **C_2_**
2	**Ia**, **Ib**	**C_2v_** $\overset{b_{1}}{\to }$ **C_s_** $\overset{a”}{\to }$ C_1_
3	**Ib**	**C_2v_** $\overset{b_{2}}{\to }$ **C_s_** $\overset{a”}{\to }$ C_1_
4	**Ic**	C_s_ $\overset{a”}{\to }$ C_1_

**Table 3:** Isohypericin model systems of symmetry groups G and the corresponding ground electronic states Γ_gr_, with DFT energies E_DFT_, JT energies E_JT_ related to the parent group G_0_, distances between hydroxyl O atoms and methyl C atoms in the 3 and 4 or the 10 and 11 positions d_OC_, and symmetries Γ_im_ and wavenumbers ν_im_ of the corresponding imaginary frequencies and resulting kernel subgroups K (G_0_, Γ_im_).

Model	G	Γ_gr_	E_DFT_ (Hartree)	E_JT_ (eV)	d_OC_ (Å)	Γ_im_	ν_im_ (cm^−1^)	K(G_0_, Γ_im_)
G_0_ = C_2h_(**IIa**)								
**IIa**	C_2h_^(a)^	X^1^A_g_	−1754.26464	0.000	2.423	b_g_	−130, −19	C_i_
						a_u_	−129, −39, −12	C_2_
**IIa**	C_i_^(b)^	X^1^A_g_	−1754.31444	1.355	2.668	-	-	-
**IIa**	C_2_^(c)^	X^1^A	−1754.31670	1.417	2.677	-	-	-
G_0_ = C_2h_(**IIb**)								
**IIb**	C_2h_^(a)^	X^1^A_g_	−1754.24601	0.000	2.636	b_g_	−380, −105, −15	C_i_
						a_u_	−379, −103, −40, −20	C_2_
**IIb**	C_i_^(b)^	X^1^A_g_	−1754.31792	1.957	2.745	-	-	-
**IIb**	C_2_^(c)^	X^1^A	−1754.31991	2.011	2.757	-	-	-
G_0_ = C_2h_(**IIc**)								
**IIc**	C_s_^(a)^	X1A’	−1754.25521	0.000	2.419 2.641	a”	−393, −133, −107, −41, −20, −15	C_1_
**IIc**	C_1_ ^(b)^	X1A	−1754.31619	1.659	2.744 2.666	-	-	-
**IIc**	C_1_ ^(c)^	X1A	−1754.31834	1.718	2.753 2.677	-	-	-

Remarks: ^(a)^ planar conformation. ^(b)^ ‘double butterfly’ conformation. ^(c)^ ‘propeller’ conformation.

**Table 4:** Symmetry descent paths of isohypericin model systems.

No.	Systems	Descent Path
5	**IIa**, **IIb**	C_2h_ $\overset{b_{g}}{\to }$ C_i_
6	**IIa**, **IIb**	C_2h_ $\overset{a_{u}}{\to }$ C_2_
7	**IIc**	C_s_ $\overset{a”}{\to }$ C_1_

**Table 5:** Fringelite D model systems of symmetry groups G and the corresponding ground electronic states Γ_gr_, with DFT energies E_DFT_, JT energies E_JT_ related to the parent group G_0_, distances between hydroxyl O atoms in the 3 and 4 (or 10 and 11) positions d_OO_, and symmetries Γ_im_ and wavenumbers ν_im_ of the corresponding imaginary frequencies and resulting kernel subgroups K (G_0_, Γ_im_).

Model	G	Γ_gr_	E_DFT_ (Hartree)	E_JT_ (eV)	d_OO_ (Å)	Γ_im_	ν_im_ (cm^−1^)	K(G_0_, Γ_im_)
G_0_ = D_2h_(**IIIa**)								
**IIIa**	D_2h_^(a)^	X^1^A_g_	−1826.09765	0.000	2.262	b_2g_	−127	C_2h_
						a_u_	−126	D_2_
						b_3u_	−37	C_2v_
**IIIa**	C_2h_^(b)^	X^1^A_g_	−1826.13780	1.093	2.516	-	-	-
**IIIa**	D_2_^(d)^	X^1^A	−1826.13963	1.142	2.526	-	-	-
**IIIa**	C_2v_^(c)^	X^1^A_1_	−1826.10503	0.201	2.344	b_2_	−94	C_s_
						a_2_	−92	C_2_
**IIIc**	C_2_^(d)^	X^1^A	−1826.15004	1.426	2.544	-	-	-
**IIId**	C_2_ ^(d)^	X1A	−1826.14935	1.407	2.551	-	-	-
**IIIc**	C_s_^(b)^	X^1^A’	−1826.14682	1.338	2.539	-	-	-
G_0_ = D_2h_(**IIIb**)								
**IIIb**	D_2h_^(a)^	X^1^A_g_	−1825.99795	0.00	2.930	b_2g_	−1466, −149	C_2h_
						a_u_	−1466, −148, −35	D_2_
						b_3g_	−659	C_2h_
						b_1u_	−657	C_2v_
						b_1g_	−654, −54	C_2h_
						b_3u_	−653, −53	C_2v_
**IIIb**	C_2h_^(b)^	X^1^A_g_	−1826.14666	4.047	2.610	-	-	-
**IIIb**	D_2_^(d)^	X^1^A	−1826.14870	4.102	2.621	-	-	-
**IIIb**	C_2v_^(c)^	X^1^A_1_	−1826.07720	2.157	2.552	a_2_	−531, −109	C_2_
						b_2_	−518, −109	C_s_
**IIIc**	C_2_^(d)^	X^1^A	−1826.15004	4.139	2.544	-	-	-
**IIId**	C_2_ ^(d)^	X^1^A	−1826.14935	4.120	2.551	-	-	-
**IIId**	C_s_^(b)^	X^1^A’	−1826.14682	4.051	2.539	-	-	-
G_0_ = C_2h_(**IIIc**)								
**IIIc**	C_2h_^(a)^	X^1^A_g_	−1826.12302	0.000	2.401	b_g_	−118,	C_i_
						a_u_	−117, −38	C_2_
**IIIc**	C_i_ ^(b)^	X^1^A_g_	−1826.14762	0.669	2.533	-	-	-
**IIIc**	C_2_^(d)^	X^1^A	−1826.15004	0.735	2.544	-	-	-
G_0_ = C_2h_(**IIId**)								
**IIId**	C_2v_^(a)^	X^1^A_1_	−1826.12138	0.000	2.406	b_1_	−119, −38	C_s_
						a_2_	−118, −11	C_2_
**IIId**	C_s_^(b)^	X^1^A’	−1826.14682	0.692	2.539	-	-	-
**IIId**	C_2_ ^(d)^	X^1^A	−1826.14935	0.761	2.551	-	-	-

Remarks: ^(a)^ planar conformation. ^(b)^ ‘double butterfly’ conformation. ^(c)^ ‘saddle’ conformation. ^(d)^ ‘propeller’ conformation.

**Table 6:** Symmetry descent paths of fringelite D (III) model systems. Possible PJT descent paths are in bold.

No.	Systems	Descent Path
8	**IIIa**, **IIIb**	**D_2h_** $\overset{b_{2g}}{\to }$ **C_2h_**
9	**IIIa**, **IIIb**	D_2h_ $\overset{a_{u}}{\to }$ D_2_
10a	**IIIa**, **IIIb**	**D_2h_** $\overset{b_{3u}}{\to }$ **C_2v_** $\overset{b_{2}}{\to }$ C_s_
10b	**IIIa**, **IIIb**	**D_2h_** $\overset{b_{3u}}{\to }$ **C_2v_** $\overset{a_{2}}{\to }$ **C_2_**
11	**IIIb**	**D_2h_** $\overset{b_{3g}}{\to }$ **C_2h_**
12a	**IIIb**	**D_2h_** $\overset{b_{1u}}{\to }$ **C_2v_** $\overset{b_{2}}{\to }$ C_s_
12b	**IIIb**	**D_2h_** $\overset{b_{1u}}{\to }$ **C_2v_** $\overset{a_{2}}{\to }$ **C_2_**
13	**IIIb**	**D_2h_** $\overset{b_{1g}}{\to }$ **C_2h_**
14a	**IIIb**	**D_2h_** $\overset{b_{3u}}{\to }$ **C_2v_** $\overset{b_{2}}{\to }$ C_s_
14b	**IIIb**	**D_2h_** $\overset{b_{3u}}{\to }$ **C_2v_** $\overset{a_{2}}{\to }$ **C_2_**
15	**IIIc**	C_2h_ $\overset{b_{g}}{\to }$ C_i_
16	**IIIc**	C_2h_ $\overset{a_{u}}{\to }$ C_2_
17	**IIId**	C_2v_ $\overset{b_{1}}{\to }$ C_s_
18	**IIId**	C_2v_ $\overset{a_{2}}{\to }$ C_2_

## Data Availability

Data are contained within the article or Supplementary Materials.

## Supplementary Materials

The following supporting information can be downloaded at: https://www.mdpi.com/article/10.3390/molecules29235624/s1, Tables S1–S3: Correlation tables for the symmetry descent of the D_2h_, C_2h_ and C_2v_ groups to their immediate subgroups; Tables S4, S6 and S8: Dipole moments, frontier orbital energies E_HOMO_, E_LUMO_ and their difference in hypericin, isohypericin and fringelite D model systems of various symmetry groups; Tables S5, S7 and S9: Symmetry groups G, the corresponding ground state symmetries Γ_gr_, the low excited state symmetries Γ_exc_ and excitation energies E_exc_, the corresponding oscillator strengths f and electron transition descriptions of hypericin, isohypericin and fringelite D model systems; Tables S10–S28: Atom numbers and coordinates (in Å) of stable compounds under study.

## References

[1] Pandey M., Pandey C., Ahuja R., Kumar R. (2023). Straining techniques for strain engineering of 2D materials towards flexible straintronic applications. Nano Energy.

[2] Bandyopadhyay S., Atulasimha J., Barman A. (2021). Magnetic straintronics: Manipulating the magnetization of magnetostrictive nanomagnets with strain for energy-efficient applications. Appl. Phys. Rev..

[3] Li T., Bandari V.K., Schmidt O.G. (2023). Molecular Electronics: Creating and Bridging Molecular Junctions and Promoting Its Commercialization. Adv. Mater..

[4] Woo S., Saka S.K., Xuan F., Yin P. (2024). Molecular robotic agents that survey molecular landscapes for information retrieval. Nat. Commun..

[5] Murali M., Gowtham H.G., Shilpa N., Krishnappa H.K.N., Ledesma A.E., Jain A.S., Shati A.A., Alfaifi M.Y., Elbehairi S.E.I., Achar R.R. (2022). Exploration of Anti-HIV Phytocompounds against SARS-CoV-2 Main Protease: Structure-Based Screening, Molecular Simulation, ADME Analysis and Conceptual DFT Studies. Molecules.

[6] Freeman D., Frolow F., Kapinus E., Lavie D., Lavie G., Merueloc D., Mazur Y. (1994). Acidic Properties of Hypericin and its Octahydroxy Analogue in the Ground and Excited States. J. Chem. Soc. Chem. Commun..

[7] Petrich J.W., Gordon M.S., Cagle M. (1998). Structure and Energetics of Ground-State Hypericin: Comparison of Experiment and Theory. J. Phys. Chem. A.

[8] Uličný J., Laaksonen A. (2000). Hypericin, an intriguing internally heterogenous molecule, forms a covalent intramolecular hydrogen bond. Chem. Phys. Lett..

[9] Guedes R.C., Eriksson L.A. (2005). Theoretical study of hypericin. J. Photochem. Photobiol. A.

[10] Shen L., Ji H.-F., Zhang H.-Y. (2006). Anion of hypericin is crucial to understanding the photosensitive features of the pigment. Bioorg. Med. Chem. Lett..

[11] Guedes R.C., Eriksson L.A. (2007). Photophysics, photochemistry, and reactivity: Molecular aspects of perylenequinone reactions. Photochem. Photobiol. Sci..

[12] Shoaf A.L., Bayse C.A. (2016). TD-DFT and structural investigation of natural photosensitive phenanthroperylene quinone derivatives. N. J. Chem..

[13] Szymanski S., Majerz I. (2019). Aromaticity and Electron Density of Hypericin. J. Nat. Prod..

[14] Siskos M.G., Choudhary M.I., Tzakos A.G., Gerothanassis I.P. (2016). ^1^H NMR chemical shift assignment, structure and conformational elucidation of hypericin with the use of DFT calculations. The challenge of accurate positions of labile hydrogens. Tetrahedron.

[15] Cvetanovic Zobenica K., Lacnjevac U., Etinski M., Vasiljevic-Radovica D., Stanisavljev D. (2019). Influence of the electron donor properties of hypericin on its sensitizing ability in DSSCs. Photochem. Photobiol. Sci..

[16] Liu Q., Wackenhut F., Hauler O., Scholz M., zur Oven-Krockhaus S., Ritz R., Adam P.-M., Brecht M., Meixner A.J. (2020). Hypericin: Single Molecule Spectroscopy of an Active Natural Drug. J. Phys. Chem. A.

[17] Liu Q., Wackenhut F., Wang L., Hauler O., Roldao J.C., Adam P.-M., Brecht M., Gierschner J., Meixner A.J. (2021). Direct Observation of Structural Heterogeneity and Tautomerization of Single Hypericin Molecules. J. Phys. Chem. Lett..

[18] De Simone B.C., Mazzone G., Toscano M., Russo N. (2022). On the origin of photodynamic activity of hypericin and its iodine-containing derivatives. J. Comput. Chem..

[19] Liu Q., Wang L., Roldao J.C., Adam P.-M., Brecht M., Gierschner J., Wackenhut F., Meixner A.J. (2021). Theoretical and Experimental Evidence of Two-Step Tautomerization in Hypericin. Adv. Photonics Res..

[20] Peeters S., Losi G., Loehlé S., Righi M.C. (2023). Aromatic molecules as sustainable lubricants explored by ab initio simulations. Carbon.

[21] Chen W.-P., Wang R.-Q., Zhang Y.-R., Song K., Tian Y., Li J.-X., Wang G.-Y., Shi G.-F. (2023). HPLC, fluorescence spectroscopy, UV spectroscopy and DFT calculations on the mechanism of scavenging •OH radicals by Hypericin. J. Mol. Struct..

[22] Bersuker I.B. (2006). The Jahn-Teller Effect.

[23] Bersuker I.B. (2013). Pseudo-Jahn−Teller Effect-A Two-State Paradigm in Formation, Deformation, and Transformation of Molecular Systems and Solids. Chem. Rev..

[24] Lewars E.G. (2016). Computational Chemistry. Introduction to the Theory and Applications of Molecular and Quantum Mechanics.

[25] Murray-Rust P., Burgi H.-B., Dunitz J.D. (1975). Description of Molecular Distortions in Terms of Symmetry Coordinates. Acta Cryst..

[26] Ceulemans A., Beyens D., Vanquickenborne L.G. (1984). Symmetry aspects of Jahn-Teller activity: Structure and reactivity. J. Am. Chem. Soc..

[27] Jahn H.A., Teller E. (1937). Stability of polyatomic molecules in degenerate electronic states. I. Orbital degeneracy. Proc. R. Soc. Lond. A.

[28] Opik U., Pryce M.H.L. (1957). Studies of the Jahn-Teller effect I. A survey of the static problem. Proc. R. Soc. Lond. A.

[29] Ceulemans A., Vanquickenborne L.G. (1989). The Epikernel Principle. Struct. Bond..

[30] Breza M., Atanasov M., Daul C., Tregenna-Piggott P.L.W. (2012). Group-Theoretical Treatment of Pseudo-Jahn-Teller Systems. Vibronic Interactions and the Jahn-Teller Effect: Theory and Application. (Prog. Theor. Chem. Phys. B 23).

[31] Salthouse J.A., Ware M.J. (1972). Point Group Character Tables and Related Data.

[32] Zhao Y., Truhlar D.G. (2008). The M06 suite of density functionals for main group thermochemistry, thermochemical kinetics, noncovalent interactions, excited states, and transition elements: Two new functionals and systematic testing of four M06-class functionals and 12 other function. Theor. Chem. Acc..

[33] Frisch G.W., Trucks M.J., Schlegel B., Scuseria G.E., Robb M.A., Cheeseman J.R., Scalmani G., Barone V., Petersson G.A., Nakatsuji H. (2016). Gaussian 16, Revision B.01.

[34] Bauernschmitt R., Ahlrichs R. (1996). Treatment of electronic excitations within the adiabatic approximation of time dependent density functional theory. Chem. Phys. Lett..

[35] Scalmani G., Frisch M.J., Mennucci B., Tomasi J., Cammi R., Barone V. (2006). Geometries and properties of excited states in the gas phase and in solution: Theory and application of a time-dependent density functional theory polarizable continuum model. J. Chem. Phys..

[36] Ugliengo P. (2012). MOLDRAW: A Program to Display and Manipulate Molecular and Crystal Structures, University Torino, Torino. https://moldraw.software.informer.com.

[37] Bersuker I.B. (The University of Texas at Austin, Austin, TX, USA), Private communication, 2024.

