# Supplementary material for: Pseudo-Jahn–Teller Effect in Natural Compounds and Its Possible Role in Straintronics I: Hypericin and Its Analogs
Source: Molecules. 2024 Nov 28;29(23):5624. doi: 10.3390/molecules29235624 (PMC11643763; doi:10.3390/molecules29235624)
Supplement: Supplementary file 1 [file molecules-29-05624-s001.zip › molecules-3302637-supplementary.pdf]

Pseudo-Jahn-Teller effect in natural compounds and its possible role in straintronics I: Hypericin and its analogs.

Dagmar Štellerová, Vladimír Lukeš, Martin Breza*

Institute of Physical Chemistry and Chemical Physics, Slovak University of Technology in Bratislava, Radlinského 9, SK-812 37 Bratislava, Slovakia

Supplementary Information

Table S1. Correlation table for the symmetry descent of the D_{2h} group to its immediate subgroups [S1]. The preserved symmetry elements are in parentheses.

Table S2. Correlation table for the symmetry descent of the C_{2h} group to its immediate subgroups [S1].

Table S3. Correlation table for the symmetry descent of the C_{2v} group to its immediate subgroups [S1]. The preserved symmetry elements are in parentheses.

Table S4. Dipole moments, frontier orbital energies E_{HOMO}, E_{LUMO} and their difference in hypericin model systems of various symmetry groups.

Table S5. Symmetry groups G, the corresponding ground state symmetries Γ_{gr}, the low excited state symmetries Γ_{exc} and excitation energies E_{exc}, the corresponding oscillator strengths f and electron transition descriptions of hypericin model systems (H = HOMO, L = LUMO).

Table S6. Dipole moments, frontier orbital energies E_{HOMO}, E_{LUMO} and their difference in isohypericin model systems of various symmetry groups.

Table S7. Symmetry groups G, the corresponding ground state symmetries Γ_{gr}, the low excited state symmetries Γ_{exc} and excitation energies E_{exc}, the corresponding oscillator strengths f and electron transition descriptions of isohypericin model systems (H = HOMO, L = LUMO).

Table S8. Dipole moments, frontier orbital energies E_{HOMO}, E_{LUMO} and their difference in fringelite D model systems of various symmetry groups.

Table S9. Symmetry groups G, the corresponding ground state symmetries Γ_{gr}, the low excited state symmetries Γ_{exc} and excitation energies E_{exc}, the corresponding oscillator strengths f and electron transition descriptions of fringelite D model systems (H = HOMO, L = LUMO).

Table S10. Atom numbers and coordinates (in Å) of stable hypericin **Ia**, C_2 symmetry 'double-butterfly'.

Table S11. Atom numbers and coordinates (in Å) of stable hypericin **Ib**, C_2 symmetry 'double-butterfly'.

Table S12. Atom numbers and coordinates (in Å) of stable hypericin **Ic**, C_1 symmetry 'propeller'.

Table S13. Atom numbers and coordinates (in Å) of stable hypericin **Ic**, C_1 symmetry 'double-butterfly'.

Table S14. Atom numbers and coordinates (in Å) of stable isohypericin **IIa**, C_i symmetry 'double-butterfly'.

Table S15. Atom numbers and coordinates (in Å) of stable isohypericin **IIa**, C_2 symmetry 'propeller'.

Table S16. Atom numbers and coordinates (in Å) of stable isohypericin **IIb**, C_i symmetry 'double-butterfly'.

Table S17. Atom numbers and coordinates (in Å) of stable isohypericin **IIb**, C_2 symmetry 'propeller'.

Table S18. Atom numbers and coordinates (in Å) of stable isohypericin **IIc**, C_1 symmetry 'double-butterfly'.

Table S19. Atom numbers and coordinates (in Å) of stable isohypericin **IIc**, C_1 symmetry 'propeller'.

Table S20. Atom numbers and coordinates (in Å) of stable fringelite D **IIIa**, C_{2h} symmetry 'double-butterfly'.

Table S21. Atom numbers and coordinates (in Å) of stable fringelite D **IIIa**, D_2 symmetry 'propeller'.

Table S22. Atom numbers and coordinates (in Å) of stable fringelite D **IIIb**, C_{2h} symmetry 'double-butterfly'.

Table S23. Atom numbers and coordinates (in Å) of stable fringelite D **IIIb**, D_2 symmetry 'propeller'.

Table S24. Atom numbers and coordinates (in Å) of stable fringelite D **IIIc**, C_2 symmetry 'propeller'.

Table S25. Atom numbers and coordinates (in Å) of stable fringelite D **IIIc**, C_s symmetry 'double-butterfly'.

Table S26. Atom numbers and coordinates (in Å) of stable fringelite D **IIIc**, C_i symmetry 'double-butterfly'.

Table S27. Atom numbers and coordinates (in Å) of stable fringelite D **IIId**, C_2 symmetry 'propeller'.

Table S28. Atom numbers and coordinates (in Å) of stable fringelite D **IIId**, C_s symmetry 'double-butterfly'.

References

Table S1. Correlation table for the symmetry descent of the D_{2h} group to its immediate subgroups [S1]. The preserved symmetry elements are in parentheses.

D_{2h}	D_2	C_{2h} (y)	C_{2v} (x)	C_{2v} (z)
A_g	A	A_g	A_1	A_1
B_{1g}	B_1	B_g	B_1	A_2
B_{2g}	B_2	A_g	B_2	B_1
B_{3g}	B_3	B_g	A_2	B_2
A_u	A	A_u	A_2	A_2
B_{1u}	B_1	B_u	B_2	A_1
B_{2u}	B_2	A_u	B_1	B_2
B_{3u}	B_3	B_u	A_1	B_1

Table S2. Correlation table for the symmetry descent of the C_{2h} group to its immediate subgroups [S1].

C_{2h}	C_2	C_s	C_i
A_g	A	A′	A_g
B_g	B	A″	A_g
A_u	A	A″	A_u
B_u	B	A′	A_u

Table S3. Correlation table for the symmetry descent of the C_{2v} group to its immediate subgroups [S1]. The preserved symmetry elements are in parentheses.

C_{2v}	C_2	C_s (yz)	C_s (xz)
A_1	A	A′	A′
A_2	A	A″	A″
B_1	B	A″	A′
B_2	B	A′	A″

Table S4. Dipole moments, frontier orbital energies E_{HOMO}, E_{LUMO} and their difference in hypericin model systems of various symmetry groups.

Model	Group	Dipole [D]	E_{HOMO} [eV]	E_{LUMO} [eV]	E_{LUMO}-E_{HOMO} [eV]
**Ia**	C_{2v} [a)]	1.279	-0.241	-0.083	0.158
**Ia**	C_2 [b)]	1.946	-0.241	-0.084	0.157
**Ia**	C_s [c)]	3.390	-0.239	-0.077	0.162
**Ib**	C_{2v} [a)]	1.641	-0.249	-0.091	0.158
**Ib**	C_2 [b)]	2.197	-0.250	-0.094	0.157
**Ib**	C_s [c)]	5.435	-0.246	-0.083	0.163
**Ic**	C_s [a)]	3.450	-0.245	-0.088	0.157
**Ic**	C_1 [b)]	2.944	-0.247	-0.090	0.157
**Ic**	C_1 [d)]	2.932	-0.247	-0.090	0.157

Remarks:

[a)]planar conformation

[b)]'double butterfly' conformation

[c)]'saddle' conformation

[d)]'propeller' conformation

Table S5. Symmetry groups G, the corresponding ground state symmetries Γ_{gr}, the low excited state symmetries Γ_{exc} and excitation energies E_{exc}, the corresponding oscillator strengths f and electron transition descriptions of hypericin model systems (H = HOMO, L = LUMO).

		**Ia** model systems				**Ib** model systems			
G	Γ_{gr}	Γ_{exc}	E_{exc} [eV]	f	Electron transitions	Γ_{exc}	E_{exc} [eV]	f	Electron transitions
C_{2v} a)	X^1A_1	1^1A_1	2.175	0.36	H→L	1^1A_1	2.709	0.35	H→L
		2^1A_1	3.554	0.00	H-2→L	1^1B_2	3.486	0.22	H-1→L
		1^1B_2	3.555	0.17	H-1→L	2^1A_1	3.518	0.00	H-2→L
		2^1B_2	3.574	0.00	H→L+1	2^1B_2	3.589	0.01	H→L+1
		3^1A_1	3.959	0.03	H-3→L	3^1A_1	3.913	0.02	H-3→L
		3^1B_2	4.047	0.01	H-4→L	3^1B_2	4.050	0.01	H-4→L
		1^1B_1	4.154	0.00	H-7→L	1^1B_1	4.063	0.00	H-7→L
		1^1A_2	4.178	0.00	H-9→L	1^1A_2	4.088	0.00	H-9→L
		4^1B_2	4.363	0.76	H→L+2	4^1B_2	4.388	0.52	H→L+2<br>H-2→L+1
		5^1B_2	4.442	0.00	H-2→L+1	5^1B_2	4.432	0.18	H→L+2
C_2 b)	X^1A	1^1A	2.667	0.34	H→L	1^1A	2.648	0.33	H→L
		1^1B	3.386	0.27	H-1→L	1^1B	3.333	0.29	H-1→L
		2^1A	3.444	0.00	H-2→L	2^1A	3.402	0.00	H-2→L
		2^1B	3.635	0.00	H-3→L	2^1B	3.639	0.00	H-3→L
		3^1A	3.939	0.03	H-4→L	3^1A	3.904	0.02	H-4→L
		3^1B	3.959	0.01	H→L+1	3^1B	3.964	0.01	H→L+1
		4^1B	4.135	0.00	H-8→L	4^1B	4.078	0.00	H-8→L
		4^1A	4.195	0.00	H-9→L	4^1A	4.139	0.00	H-9→L
		51B	4.403	0.22	H-2→L+1	5^1B	4.400	0.12	H-5→L
		51A	4.460	0.00	H-1→L+1	5^1A	4.450	0.00	H-1→L+1
C_s c)	X^1A'	$1^1A'$	2.785	0.35	H→L	$1^1A'$	2.795	0.33	H→L
		$2^1A'$	3.549	0.01	H-1→L	$1^1A''$	3.542	0.15	H-1→L
		$1^1A''$	3.564	0.14	H-2→L	$2^1A'$	3.546	0.01	H-2→L
		$2^1A''$	3.576	0.01	H→L+1	$2^1A''$	3.566	0.01	H→L+1
		$3^1A'$	4.003	0.04	H-3→L	$3^1A'$	4.022	0.04	H-3→L
		$3^1A''$	4.072	0.01	H-4→L	$3^1A''$	4.132	0.01	H-4→L
		$4^1A'$	4.214	0.00	H-8→L	$4^1A'$	4.150	0.00	H-8→L
		$4^1A''$	4.240	0.00	H-9→L	$4^1A''$	4.176	0.00	H-9→L
		$5^1A''$	4.324	0.39	H-1→L+1	$5^1A''$	4.258	0.37	H-2→L+1
		$6^1A''$	4.399	0.39	H→L+2	$5^1A'$	4.352	0.03	H-1→L+1

Table S5. (cont.)

G	Γ_{gr}	Γ_{exc}	E_{exc} [eV]	f	Electron transitions
		**Ic** model systems			
C_s [a)]	X^1A'	$1^1A'$	2.684	0.34	H→L
		$2^1A'$	3.404	0.12	H-1→L
		$3^1A'$	3.546	0.05	H-2→L
		$4^1A'$	3.589	0.01	H→L+1
		$5^1A'$	3.858	0.01	H-3→L
		$1^1A''$	4.114	0.00	H-6→L
		$6^1A'$	4.116	0.05	H-4→L
		$2^1A''$	4.141	0.00	H-9→L
		$7^1A'$	4.325	0.55	H→L+2
		$8^1A'$	4.414	0.02	H-2→L+1
					H-1→L+1
C_1 [b)]	X^1A	1^1A	2.648	0.33	H→L
		2^1A	3.325	0.24	H-1→L
		3^1A	3.422	0.03	H-2→L
		4^1A	3.631	0.00	H-3→L
		5^1A	3.868	0.01	H-3→L
		6^1A	4.025	0.04	H-4→L
		7^1A	4.094	0.00	H-8→L
		8^1A	4.153	0.00	H-9→L
		9^1A	4.393	0.13	H-5→L
					H→L+2
		10^1A	4.461	0.01	H-1→L+1
C_1 [d)]	X^1A	1^1A	2.647	0.32	H→L
		2^1A	3.298	0.25	H-1→L
		3^1A	3.399	0.03	H-2→L
		4^1A	3.633	0.01	H-3→L
		5^1A	3.870	0.02	H→L+1
					H-3→L
		6^1A	4.027	0.03	H-4→L
		7^1A	4.106	0.00	H-8→L
		8^1A	4.142	0.00	H-9→L
		9^1A	4.382	0.13	H-5→L
		10^1A	4.450	0.00	H-1→L+1

Remarks:

[a)]planar conformation

[b)]'double butterfly' conformation

[c)]'saddle' conformation

[d)]'propeller' conformation

Table S6. Dipole moments, frontier orbital energies E_{HOMO}, E_{LUMO} and their difference in isohypericin model systems of various symmetry groups

Model	Group	Dipole [D]	E_{HOMO} [eV]	E_{LUMO} [eV]	E_{LUMO}-E_{HOMO} [eV]
**IIa**	C_{2h} [a)]	0.000	-0.241	-0.083	0.158
**IIa**	C_2 [c)]	0.418	-0.242	-0.084	0.158
**IIa**	C_i [b)]	0.000	-0.242	-0.084	0.157
**IIb**	C_{2h} [a)]	0.000	-0.247	-0.090	0.157
**IIb**	C_2 [c)]	0.674	-0.251	-0.094	0.157
**IIb**	C_i [b)]	0.000	-0.250	-0.094	0.157
**IIc**	C_s [a)]	3.358	-0.244	-0.087	0.157
**IIc**	C_1 [b)]	2.937	-0.246	-0.089	0.157
**IIc**	C_1 [c)]	2.895	-0.246	-0.089	0.157

Remarks:

[a)]planar conformation

[b)]'double butterfly' conformation

[c)]'propeller' conformation

Table S7. Symmetry groups G, the corresponding ground state symmetries Γ_{gr}, the low excited state symmetries Γ_{exc} and excitation energies E_{exc}, the corresponding oscillator strengths f and electron transition descriptions of isohypericin model systems (H = HOMO, L = LUMO).

		**IIa** model systems				**IIb** model systems			
G	Γ_{gr}	Γ_{exc}	E_{exc} [eV]	f	Electron transitions	Γ_{exc}	E_{exc} [eV]	f	Electron transitions
C_{2h} a)	X^1A_g	1^1B_u	2.719	0.37	H→L	1^1B_u	2.702	0.37	H→L
		1^1A_g	3.560	0.00	H-1→L	1^1A_g	3.471	0.00	H-2→L
		2^1B_u	3.583	0.17	H-2→L	2^1B_u	3.475	0.18	H-1→L
		2^1A_g	3.596	0.00	H→L+1	2^1A_g	3.607	0.00	H→L+1
		3^1B_u	3.966	0.03	H-3→L	3^1B_u	3.930	0.02	H-4→L
		3^1A_g	4.076	0.00	H-4→L	3^1A_g	3.985	0.00	H-3→L
		1^1B_g	4.161	0.00	H-8→L	1^1B_g	4.107	0.00	H-6→L
		1^1A_u	4.185	0.00	H-9→L	1^1A_u	4.132	0.00	H-9→L
		4^1B_u	4.379	0.80	H→L+2	4^1B_u	4.361	0.74	H→L+2
		5^1B_u	4.458	0.01	H-1→L+1	4^1A_g	4.418	0.00	H-1→L+1
C_2 c)	X^1A	1^1B	2.667	0.34	H→L	1^1B	2.640	0.33	H→L
		2^1B	3.871	0.27	H-1→L	2^1B	3.322	0.27	H-1→L
		1^1A	3.434	0.00	H-2→L	1^1A	3.357	0.00	H-2→L
		2^1A	3.652	0.02	H-3→L	2^1A	3.640	0.01	H-3→L
		3^1B	3.946	0.03	H-4→L	3^1B	3.893	0.02	H-4→L
		3^1A	3.986	0.00	H→L+1	3^1A	3.962	0.01	H→L+1
		4^1B	4.155	0.00	H-8→L	4^1B	4.101	0.00	H-8→L
		4^1A	4.174	0.00	H-9→L	4^1A	4.121	0.00	H-9→L
		5^1B	4.232	0.37	H-2→L+1	5^1B	4.389	0.32	H-2→L+1
		5^1A	4.522	0.00	H-1→L+1	5^1A	4.399	0.00	H-5→L
C_i b)	X^1A_g	1^1A_u	2.673	0.35	H→L	1^1A_u	2.647	0.34	H→L
		2^1A_u	3.418	0.27	H-1→L	2^1A_u	3.352	0.28	H-1→L
		1^1A_g	3.456	0.00	H-2→L	1^1A_g	3.377	0.00	H-2→L
		2^1A_g	3.646	0.00	H-3→L<br>H→L+1	2^1A_g	3.635	0.00	H-3→L
		3^1A_u	3.948	0.03	H-4→L	3^1A_u	3.897	0.02	H-4→L
		3^1A_g	3.986	0.00	H-3→L<br>H→L+1	3^1A_g	3.962	0.00	H→L+1<br>H-3→L
		4^1A_g	4.134	0.00	H-8→L	4^1A_g	4.078	0.00	H-8→L
		4^1A_u	4.193	0.00	H-9→L	4^1A_u	4.142	0.00	H-9→L
		5^1A_u	4.440	0.36	H-2→L+1	5^1A_u	4.407	0.30	H-2→L+1
		5^1A_g	4.460	0.00	H-1→L+1	5^1A_g	4.409	0.00	H-1→L+1

Table S7. (cont.)

		**IIc** model systems			
G	Γ_{gr}	Γ_{exc}	E_{exc} [eV]	f	Electron transitions
C_s [a)]	X^1A'	$1^1A'$	2.700	0.36	H→L
		$2^1A'$	3.487	0.07	H-1→L
		$2^1A'$	3.548	0.11	H-2→L
		$4^1A'$	3.597	0.00	H→L+1
		$5^1A'$	3.890	0.00	H-3→L
		$6^1A'$	4.082	0.03	H-4→L
		$1^1A''$	4.123	0.00	H-7→L
		$2^1A''$	4.166	0.00	H-9→L
		$7^1A'$	4.350	0.70	H→L+2
		$8^1A'$	4.446	0.01	H-2→L+1<br>H-1→L+1
C_1 [b)]	X^1A	1^1A	2.656	0.34	H→L
		2^1A	3.383	0.27	H-1→L
		3^1A	3.416	0.01	H-2→L
		4^1A	3.634	0.00	H-3→L<br>H→L+1
		5^1A	3.894	0.01	H-4→L<br>H-3→L
		6^1A	4.002	0.02	H-4→L<br>H→L+1
		7^1A	4.104	0.00	H-8→L<br>H-5→L
		8^1A	4.169	0.00	H-9→L<br>H-8→L+1
		9^1A	4.417	0.27	H-2→L+1
		10^1A	4.438	0.05	H-1→L+1<br>H-5→L
C_1 [c)]	X^1A	1^1A	2.650	0.33	H→L
		2^1A	3.352	0.27	H-1→L
		3^1A	3.394	0.00	H-2→L
		4^1A	3.639	0.01	H-3→L
		5^1A	3.894	0.01	H-4→L
		6^1A	4.002	0.02	H-4→L<br>H→L+1
		7^1A	4.121	0.00	H-8→L
		8^1A	4.152	0.00	H-9→L
		9^1A	4.401	0.32	H-2→L<br>H→L+2
		10^1A	4.427	0.03	H→L+1<br>H-5→L

Remarks:

[a)]planar conformation

[b)]'double butterfly' conformation

[c)]'propeller' conformation

Table S8. Dipole moments, frontier orbital energies E_{HOMO}, E_{LUMO} and their difference in fringelite D model systems of various symmetry groups

Model	Group	Dipole [D]	E_{HOMO} [eV]	E_{LUMO} [eV]	E_{LUMO} - E_{HOMO} [eV]
**IIIa**	D_{2h} a)	0.000	-0.238	-0.082	0.156
**IIIa**	D_2 d)	0.000	-0.237	-0.079	0.158
**IIIa**	C_{2h} b)	0.000	-0.237	-0.078	0.158
**IIIa**	C_{2v} s)	2.384	-0.236	-0.077	0.160
**IIIb**	D_{2h} a)	0.000	-0.254	-0.098	0.157
**IIIb**	D_2 d)	0.000	-0.256	-0.098	0.157
**IIIb**	C_{2h} b)	0.000	-0.255	-0.098	0.157
**IIIb**	C_{2v} c)	6.059	-0.250	-0.088	0.162
**IIIc**	C_{2h} a)	0.000	-0.246	-0.090	0.156
**IIIc**	C_i b)	0.000	-0.248	-0.090	0.158
**IIIc**	C_2 d)	3.123	-0.248	-0.090	0.158
**IIId**	C_{2v} a)	6.709	-0.244	-0.091	0.153
**IIId**	C_s b)	5.812	-0.247	-0.091	0.156
**IIId**	C_2 d)	4.867	-0.248	-0.091	0.157

Remarks:

a)planar conformation

b)'double butterfly' conformation

c)'saddle' conformation

d)'propeller' conformation

Table S9. Symmetry groups G, the corresponding ground state symmetries Γ_{gr}, the low excited state symmetries Γ_{exc} and excitation energies E_{exc}, the corresponding oscillator strengths f and electron transition descriptions of fringelite D model systems (H = HOMO, L = LUMO).

		**IIIa** model systems				**IIIb** model systems			
G	Γ_{gr}	Γ_{exc}	E_{exc} [eV]	f	Electron transitions	Γ_{exc}	E_{exc} [eV]	f	Electron transitions
D_{2h} a)	X^1A_g	1^1B_{2u}	2.670	0.34	H→L	1^1B_{2u}	2.671	0.32	H→L
		1^1A_g	3.554	0.00	H-1→L	1^1B_{3u}	3.438	0.25	H-1→L
		1^1B_{1u}	3.564	0.13	H-2→L	1^1A_g	3.474	0.00	H-2→L
		1^1B_{3g}	3.596	0.00	H→L+1<br>H-3→L	1^1B_{1g}	3.608	0.00	H-4→L<br>H→L+1
		2^1B_{2u}	3.864	0.01	H-4→L	2^1B_{2u}	3.765	0.00	H-3→L
		2^1B_{3g}	3.978	0.00	H-3→L	2^1B_{1g}	3.963	0.00	H→L+1<br>H-4→L
		1^1B_{1g}	4.288	0.00	H-8→L	1^1B_{3g}	4.102	0.00	H-7→L
		1^1A_u	4.313	0.00	H-9→L	1^1A_u	4.128	0.00	H-9→L
		2^1B_{1u}	4.319	0.82	H→L+2	2^1B_{3u}	4.390	0.67	H→L+2
		3^1B_{3g}	4.581	0.00	H-6→L	3^1B_{1g}	4.486	0.00	H-5→L
D_2 d)	X^1A	1^1B_2	2.683	0.31	H→L	1^1B_2	2.648	0.29	H→L
		1^1B_1	3.476	0.22	H-1→L	1^1B_3	3.370	0.26	H-1→L
		1^1A	3.493	0.00	H-2→L	1^1A	3.398	0.00	H-2→L
		1^1B_3	3.610	0.01	H-3→L	1^1B_1	3.590	0.01	H-3→L
		2^1B_2	3.930	0.02	H-4→L	2^1B_2	3.852	0.01	H-4→L
		2^1B_3	4.001	0.01	H→L+1	2^1B_1	4.021	0.01	H→L+1
		2^1B_1	4.281	0.04	H-8→L	2^1B_3	4.164	0.00	H-8→L
		2^1A	4.308	0.00	H-9→L	2^1A	4.192	0.00	H-9→L
		3^1B_1	4.398	0.74	H→L+2	3^1B_3	4.406	0.69	H→L+2
		3^1B_3	4.559	0.00	H-5→L	3^1B_1	4.474	0.00	H-5→L
C_{2h} b)	X^1A_g	1^1A_u	2.691	0.32	H→L	1^1A_u	2.653	0.30	H→L
		1^1B_u	3.503	0.22	H-1→L	1^1B_u	3.394	0.26	H-1→L
		1^1A_g	3.516	0.00	H-2→L	1^1A_g	3.420	0.00	H-2→L
		1^1B_g	3.607	0.00	H-3→L	1^1B_g	3.585	0.00	H-3→L
		1^1A_u	3.937	0.02	H-4→L	2^1A_u	3.858	0.00	H-4→L
		2^1B_g	4.001	0.00	H→L+1	2^1B_g	4.016	0.00	H→L+1
		3^1B_g	4.271	0.00	H-8→L	3^1B_g	4.152	0.00	H-8→L
		2^1A_u	4.325	0.00	H-9→L	3^1A_u	4.208	0.00	H-9→L
		2^1B_u	4.422	0.81	H→L+2	2^1B_u	4.433	0.72	H→L+2
		2^1A_g	4.572	0.00	H-1→L+1	4^1B_g	4.489	0.00	H-5→L
C_{2v} c)	X^1A_1	1^1B_1	2.742	0.33	H→L	1^1B_2	2.784	0.32	H→L
		1^1A_1	3.565	0.01	H-1→L	1^1A_1	3.544	0.00	H-2→L
		1^1B_2	3.590	0.11	H-2→L	1^1B_1	3.548	0.15	H-1→L
		1^1A_2	3.640	0.00	H→L+1	1^1A_2	3.645	0.00	H→L+1
		1^1B_1	3.924	0.02	H-4→L	2^1B_2	3.963	0.02	H-3→L
		2^1A_2	4.025	0.00	H-3→L	2^1A_2	4.114	0.00	H-4→L
		2^1B_1	4.329	0.00	H-8→L	2^1B_2	4.192	0.00	H-8→L
		2^1B_2	4.339	0.84	H→L+2	3^1A_2	4.215	0.00	H-9→L
		3^1A_2	4.354	0.00	H-9→L	2^1B_1	4.337	0.74	H→L+2
		3^1B_2	4.562	0.04	H-1→L+1	3^1B_1	4.426	0.11	H-2→L+1

Table S9. (cont.)

	**IIIc** model systems						**IIId** model systems				
G	Γ_{gr}	Γ_{exc}	E_{exc} [eV]	f	Electron transitions	G	Γ_{gr}	Γ_{exc}	E_{exc} [eV]	f	Electron transitions
C_{2h} a)	X^1A_g	1^1B_u	2.661	0.33	H→L	C_{2v} a)	X^1A_1	1^1B_2	2.577	0.28	H→L
		2^1B_u	3.408	0.17	H-1→L			1^1A_1	3.340	0.13	H-1→L
		1^1A_g	3.418	0.00	H-2→L			2^1B_2	3.421	0.01	H-3→L
		2^1A_g	3.640	0.00	H-3→L			2^1A_1	3.517	0.05	H-2→L
					H→L+1						
		3^1B_u	3.866	0.01	H-4→L			3^1B_2	3.755	0.01	H→L+1
		3^1A_g	3.927	0.00	H-3→L			4^1B_2	4.129	0.06	H-4→L
					H→L+1						
		1^1B_g	4.220	0.00	H-8→L			1^1A_2	4.181	0.00	H-7→L
		1^1A_u	4.248	0.00	H-9→L			3^1A_1	4.229	0.54	H→L+2
		4^1B_u	4.345	0.75	H→L+2			2^1A_2	4.246	0.00	H-9→L
		3^1A_g	4.437	0.00	H-5→L			5^1B_2	4.492	0.00	H-6→L
											H→L+3
C_2 d)	X^1A	1^1B	2.683	0.31	H→L	C_2 d)	X^1A	1^1B	2.651	0.29	H→L
		2^1B	3.392	0.25	H-1→L			1^1A	3.372	0.20	H-1→L
		1^1A	3.422	0.00	H-2→L			2^1A	3.450	0.05	H-2→L
		2^1A	3.626	0.01	H-3→L			2^1B	3.513	0.01	H-3→L
		3^1B	3.921	0.01	H-4→L			3^1B	3.889	0.01	H→L+1
		3^1A	4.010	0.01	H→L+1			4^1B	4.108	0.04	H-4→L
											H→L+1
		4^1B	4.202	0.00	H-8→L			3^1A	4.172	0.00	H-8→L
											H-7→L
		4^1A	4.229	0.00	H-9→L			4^1A	4.239	0.00	H-9→L
		5^1B	4.433	0.71	H→L+2			5^1A	4.394	0.60	H→L+2
		5^1A	4.481	0.00	H-5→L			5^1B	4.512	0.00	H-5→L
C_i b)	X^1A_g	1^1A_u	2.682	0.32	H→L	C_s b)	X^1A'	$1^1A''$	2.649	0.30	H→L
		2^1A_u	3.417	0.24	H-1→L			$1^1A'$	3.397	0.20	H-1→L
		1^1A_g	3.441	0.00	H-2→L			$2^1A'$	3.470	0.05	H-2→L
		3^1A_u	3.627	0.00	H-3→L			$2^1A''$	3.514	0.00	H-3→L
		4^1A_u	3.930	0.02	H-4→L			$3^1A''$	3.882	0.00	H→L+1
		2^1A_g	3.999	0.00	H→L+1			$4^1A''$	4.104	0.04	H-4→L
		3^1A_g	4.194	0.00	H-8→L			$5^1A''$	4.170	0.00	H-8→L
		5^1A_u	4.239	0.00	H-9→L			$6^1A''$	4.245	0.00	H-9→L
		6^1A_u	4.452	0.76	H→L+2			$3^1A'$	4.410	0.63	H→L+2
		4^1A_g	4.488	0.00	H-5→L			$7^1A''$	4.517	0.00	H-5→L

Remarks:

a) planar conformation

b) 'double butterfly' conformation

c) 'saddle' conformation

d) 'propeller' conformation

Table S10. Atom numbers and coordinates (in Å) of stable hypericin **Ia**, C_2 symmetry 'double-butterfly'.

6	0.060049	-3.563824	-2.461415
8	0.170964	-4.882710	-2.521450
6	-0.265069	-2.874559	-3.637828
6	-0.386282	-1.498313	-3.631135
8	-0.921940	-0.854870	-4.682386
6	-0.035161	-0.720732	-2.477426
6	0.035161	0.720732	-2.477426
6	0.386282	1.498313	-3.631135
8	0.921940	0.854870	-4.682386
6	0.265069	2.874559	-3.637828
6	-0.060049	3.563824	-2.461415
8	-0.170964	4.882710	-2.521450
6	-0.167977	2.845443	-1.250769
6	-0.088636	1.422521	-1.259565
6	-0.263638	3.577336	-0.013038
8	-0.409776	4.834599	-0.008156
6	-0.132247	2.842835	1.230899
6	-0.071532	1.421820	1.238956
6	0.025015	3.559418	2.433169
8	-0.071532	4.882081	2.506827
6	0.407528	2.856970	3.586850
6	0.485037	1.477628	3.608031
6	0.075467	0.722654	2.460854
6	-0.075467	-0.722654	2.460854
6	-0.485037	-1.477628	3.608031
6	-0.407528	-2.856970	3.586850
6	-0.025015	-3.559418	2.433169
8	0.071532	-4.882081	2.506827
6	0.132247	-2.842835	1.230899
6	0.071532	-1.421820	1.238956

6	0.263638	-3.577336	-0.013038
8	0.409776	-4.834599	-0.008156
6	0.167977	-2.845443	-1.250769
6	0.088636	-1.422521	-1.259565
6	0.078258	-0.695356	-0.011892
6	-0.078258	0.695356	-0.011892
6	-1.170853	-0.862635	4.806839
6	1.170853	0.862635	4.806839
1	-0.497205	-3.457963	-4.530043
1	0.497205	3.457963	-4.530043
1	0.709618	3.444675	4.455106
1	-0.709618	-3.444675	4.455106
1	-0.322206	5.188325	-1.583807
1	-0.259738	5.198059	1.583986
1	0.322206	-5.188325	-1.583807
1	0.259738	-5.198059	1.583986
1	2.127814	1.382938	4.953853
1	-2.127814	-1.382938	4.953853
1	-1.125722	-1.501730	-5.371497
1	1.125722	1.501730	-5.371497
1	-1.383967	0.202670	4.671519
1	-0.585316	-0.992627	5.727016
1	1.383967	-0.202670	4.671519
1	0.585316	0.992627	5.727016

Table S11. Atom numbers and coordinates (in Å) of stable hypericin **Ib**, C_2 symmetry 'double-butterfly'.

C	0.04825195	-3.57101210	-2.46138125
O	0.15985394	-4.88972108	-2.51782870
C	-0.28282404	-2.88707269	-3.63618702
C	-0.39115803	-1.51077669	-3.63298954
O	-0.86815202	-0.97384429	-4.77936236
C	-0.04320504	-0.72434509	-2.48393527

C	0.04320504	0.72434509	-2.48393527
C	0.39115803	1.51077669	-3.63298954
O	0.86815202	0.97384429	-4.77936236
C	0.28282404	2.88707269	-3.63618702
C	-0.04825195	3.57101210	-2.46138125
O	-0.15985394	4.88972108	-2.51782870
C	-0.16117402	2.84605749	-1.25219404
C	-0.08457603	1.42608749	-1.26166153
C	-0.26085303	3.57667406	-0.00960779
O	-0.41156702	4.83144106	-0.00335436
C	-0.12878904	2.84134063	1.23180996
C	-0.06788905	1.42039863	1.23760547
C	0.02581695	3.55702921	2.43493021
O	-0.07244804	4.87812319	2.51080866
C	0.40912994	2.85384481	3.58896097
C	0.48714393	1.47557581	3.60868449
C	0.07699394	0.72144021	2.45952623
C	-0.07699394	-0.72144021	2.45952623
C	-0.48714393	-1.47557581	3.60868449
C	-0.40912994	-2.85384481	3.58896097
C	-0.02581695	-3.55702921	2.43493021
O	0.07244804	-4.87812319	2.51080866
C	0.12878904	-2.84134063	1.23180996
C	0.06788905	-1.42039863	1.23760547
C	0.26085303	-3.57667406	-0.00960779
O	0.41156702	-4.83144106	-0.00335436
C	0.16117402	-2.84605749	-1.25219404
C	0.08457603	-1.42608749	-1.26166153
C	0.07586895	-0.69560394	-0.01346326
C	-0.07586895	0.69560394	-0.01346326
C	-1.17383709	-0.85849760	4.80587068
C	1.17383709	0.85849760	4.80587068

H	-0.52920904	-3.44561838	-4.53728321
H	0.52920904	3.44561838	-4.53728321
H	0.71149294	3.44145651	4.45715417
H	-0.71149294	-3.44145651	4.45715417
H	-0.31673900	5.19635160	-1.58396723
H	-0.26192103	5.19682951	1.58932877
H	0.31673900	-5.19635160	-1.58396723
H	0.26192103	-5.19682951	1.58932877
H	2.13222092	1.37656033	4.95087547
H	-2.13222092	-1.37656033	4.95087547
H	-1.09976401	-0.04316934	-4.65239304
H	1.09976401	0.04316934	-4.65239304
H	-1.38436408	0.20723645	4.67047404
H	-0.59032610	-0.99044192	5.72696663
H	1.38436408	-0.20723645	4.67047404
H	0.59032610	0.99044192	5.72696663

Table S12. Atom numbers and coordinates (in Å) of stable hypericin **Ic**, C_1 symmetry 'propeller'.

6	-3.470897	1.182410	-2.190072
8	-4.779900	1.075393	-2.366126
6	-2.728523	1.864266	-3.155818
6	-1.369154	2.068325	-3.008584
8	-0.770482	2.597136	-4.087665
6	-0.667386	1.631280	-1.822907
6	0.737290	1.884702	-1.536855
6	1.487503	3.000269	-2.021248
8	0.808831	3.998371	-2.663161
6	2.844194	3.143640	-1.833300
6	3.556458	2.199129	-1.082787
8	4.868008	2.347034	-0.976307
6	2.839958	1.170164	-0.441421

6	1.430379	1.052803	-0.622276
6	3.568316	0.229295	0.384021
8	4.827057	0.296426	0.482145
6	2.824033	-0.789915	1.090182
6	1.402401	-0.792231	1.065798
6	3.517279	-1.778517	1.815917
8	4.841053	-1.808907	1.916779
6	2.782911	-2.829514	2.387531
6	1.400940	-2.831888	2.393162
6	0.682158	-1.710909	1.862657
6	-0.751713	-1.529031	1.999792
6	-1.507095	-1.966835	3.136172
6	-2.887571	-1.933580	3.097516
6	-3.584995	-1.425684	1.988686
8	-4.910379	-1.501183	1.990187
6	-2.853734	-0.784438	0.971697
6	-1.434401	-0.785373	1.008732
6	-3.559683	-0.140453	-0.119022
8	-4.818161	-0.232879	-0.206000
6	-2.797051	0.604538	-1.090937
6	-1.389355	0.780968	-0.948577
6	-0.693406	0.032042	0.074098
6	0.697854	0.101088	0.175006
6	-0.876331	-2.316453	4.464660
6	0.730560	-4.117503	2.819391
1	-3.218729	2.228551	-4.056352
1	3.384819	3.998494	-2.242279
1	3.342677	-3.675023	2.790064
1	-3.476461	-2.264261	3.954455
1	5.181096	1.595858	-0.397998
1	5.178075	-1.021138	1.417932
1	-5.123574	0.557228	-1.589439

1	-5.212603	-1.054543	1.155170
1	1.193344	-4.944187	2.262249
1	-1.345175	-1.693629	5.239440
1	-0.020891	3.151679	-3.813152
1	1.416119	4.727977	-2.852440
1	0.201404	-2.123739	4.483931
1	-1.053166	-3.365156	4.739574
1	-0.343012	-4.124533	2.604849
1	0.879572	-4.321924	3.888334

Table S13. Atom numbers and coordinates (in Å) of stable hypericin **Ic**, C_1 symmetry 'double-butterfly'.

6	-3.755186	2.040161	-0.574170
8	-5.069119	1.953196	-0.716629
6	-3.185732	3.310802	-0.487411
6	-1.819815	3.478983	-0.368271
8	-1.445307	4.736971	-0.085150
6	-0.914121	2.352989	-0.435887
6	0.536911	2.450549	-0.448873
6	1.274924	3.579431	-0.923889
8	0.592897	4.565631	-1.580897
6	2.639371	3.704807	-0.796555
6	3.396489	2.650427	-0.271874
8	4.702174	2.825749	-0.144159
6	2.744399	1.436758	0.024268
6	1.326679	1.326831	-0.093940
6	3.562550	0.290831	0.356509
8	4.808858	0.414875	0.534507
6	2.930614	-1.006040	0.423894
6	1.516575	-1.130090	0.334324
6	3.740049	-2.157628	0.498903
8	5.059398	-2.109216	0.637307

6	3.139334	-3.411932	0.311801
6	1.768782	-3.550737	0.215185
6	0.920093	-2.409960	0.401190
6	-0.520929	-2.497234	0.532992
6	-1.191694	-3.610149	1.138454
6	-2.563867	-3.721524	1.037965
6	-3.342034	-2.723025	0.430158
8	-4.649009	-2.925547	0.322300
6	-2.717915	-1.514925	0.063953
6	-1.306399	-1.383719	0.151098
6	-3.542220	-0.387672	-0.318897
8	-4.787082	-0.536387	-0.491922
6	-2.920493	0.906750	-0.442735
6	-1.506736	1.067726	-0.339179
6	-0.683507	-0.095461	-0.076882
6	0.697984	0.041324	0.101026
6	-0.500767	-4.604829	2.043588
6	1.261137	-4.894858	-0.254431
1	-3.827005	4.189510	-0.463159
1	3.161056	4.598982	-1.141016
1	3.798169	-4.271559	0.180491
1	-3.089934	-4.564570	1.488544
1	5.063823	1.952002	0.179267
1	5.301911	-1.147729	0.671769
1	-5.286574	0.981542	-0.699768
1	-5.028501	-2.082925	-0.046292
1	1.810550	-5.163877	-1.167682
1	-1.028690	-4.608806	3.007654
1	-0.585786	4.929080	-0.495979
1	1.224239	5.208487	-1.934523
1	0.546195	-4.348477	2.235018
1	-0.548189	-5.626516	1.643133

1	0.193102	-4.884452	-0.494201
1	1.451463	-5.685330	0.484032

Table S14. Atom numbers and coordinates (in Å) of stable isohypericin **IIa**, C_i symmetry 'double-butterfly'.

C	-3.59784284	2.39508114	0.05800810
O	-4.91987184	2.44606121	0.16961012
C	-2.91872379	3.57236711	-0.29664589
C	-1.54373779	3.62256404	-0.40179691
C	-0.96940673	4.85535102	-1.05985690
C	-0.76577884	2.46982900	-0.04326093
C	0.67882616	2.47009192	0.06038105
C	1.43477522	3.62130388	0.45019406
O	0.75478528	4.66549491	0.96701108
C	2.81343323	3.65352681	0.37151304
C	3.52652616	2.49701477	0.02639002
O	4.84598816	2.58052471	-0.06309999
C	2.82615010	1.28136181	-0.12769598
C	1.40306610	1.26585689	-0.06923297
C	3.57738903	0.05627677	-0.26026400
O	4.83457303	0.07810771	-0.40384202
C	2.86226897	-1.20009719	-0.16947701
C	1.44091197	-1.23306512	-0.09106600
C	3.59784284	-2.39508114	-0.05800810
O	4.91987184	-2.44606121	-0.16961012
C	2.91872379	-3.57236711	0.29664589
C	1.54373779	-3.62256404	0.40179691
C	0.76577884	-2.46982900	0.04326093
C	-0.67882616	-2.47009192	-0.06038105
C	-1.43477522	-3.62130388	-0.45019406
C	-2.81343323	-3.65352681	-0.37151304
C	-3.52652616	-2.49701477	-0.02639002

O	-4.84598816	-2.58052471	0.06309999
C	-2.82615010	-1.28136181	0.12769598
C	-1.40306610	-1.26585689	0.06923297
C	-3.57738903	-0.05627677	0.26026400
C	-2.86226897	1.20009719	0.16947701
C	-1.44091197	1.23306512	0.09106600
C	-0.69554297	-0.00567201	0.07743204
C	0.69554297	0.00567201	-0.07743204
O	-0.75478528	-4.66549491	-0.96701108
C	0.96940673	-4.85535102	1.05985690
H	-3.52719574	4.43823215	-0.56166888
H	3.37641227	4.54977577	0.63631405
H	3.52719574	-4.43823215	0.56166888
H	-3.37641227	-4.54977577	-0.63631405
H	5.16741911	1.65401669	-0.24611600
H	5.22188095	-1.51251331	-0.32421004
H	-5.22188095	1.51251331	0.32421004
H	-5.16741911	-1.65401669	0.24611600
H	1.61525779	-5.12456815	1.90726396
H	-1.38558926	-5.35121396	-1.22609401
H	-1.61525779	5.12456815	-1.90726396
H	1.38558926	5.35121396	1.22609401
O	-4.83457303	-0.07810771	0.40384202
H	0.04273226	4.68460097	-1.44455291
H	-0.93452368	5.70549501	-0.36806589
H	0.93452368	-5.70549501	0.36806589
H	-0.04273226	-4.68460097	1.44455291

Table S15. Atom numbers and coordinates (in Å) of stable isohypericin **IIa**, C_2 symmetry 'propeller'.

C	-3.60474962	-2.33597263	0.39836088
O	-4.93226058	-2.37184298	0.42085584

C	-2.90279343	-3.50817492	0.72741789
C	-1.52562336	-3.58384952	0.66024792
C	-0.88957212	-4.81450087	1.26203993
C	-0.77903849	-2.45521475	0.18128695
C	0.64103854	-2.47551031	-0.11149102
C	1.33104773	-3.63545761	-0.58705001
O	0.58176581	-4.68991036	-0.97029604
C	2.70602676	-3.66661921	-0.72378098
C	3.46763858	-2.51308144	-0.48850895
O	4.78637568	-2.60215109	-0.58829492
C	2.80424534	-1.29786411	-0.21863196
C	1.38614032	-1.27760355	-0.09018799
C	3.57858311	-0.08810533	-0.06323593
O	4.84251714	-0.11741604	-0.10653490
C	2.87325384	1.16097896	0.14720806
C	1.45209578	1.21406051	0.09295702
C	3.60474962	2.33597263	0.39836088
O	4.93226058	2.37184298	0.42085584
C	2.90279343	3.50817492	0.72741789
C	1.52562336	3.58384952	0.66024792
C	0.77903849	2.45521475	0.18128695
C	-0.64103854	2.47551031	-0.11149102
C	-1.33104773	3.63545761	-0.58705001
C	-2.70602676	3.66661921	-0.72378098
C	-3.46763858	2.51308144	-0.48850895
O	-4.78637568	2.60215109	-0.58829492
C	-2.80424534	1.29786411	-0.21863196
C	-1.38614032	1.27760355	-0.09018799
C	-3.57858311	0.08810533	-0.06323593
O	-4.84251714	0.11741604	-0.10653490
C	-2.87325384	-1.16097896	0.14720806
C	-1.45209578	-1.21406051	0.09295702

C	-0.69831499	0.01309933	0.00666496
C	0.69831499	-0.01309933	0.00666496
O	-0.58176581	4.68991036	-0.97029604
C	0.88957212	4.81450087	1.26203993
H	-3.48674058	-4.35544951	1.08981887
H	3.22080569	-4.56749168	-1.06111097
H	3.48674058	4.35544951	1.08981887
H	-3.22080569	4.56749168	-1.06111097
H	5.13650474	-1.68359574	-0.42170690
H	5.23869454	1.45064136	0.21416512
H	-5.23869454	-1.45064136	0.21416512
H	-5.13650474	1.68359574	-0.42170690
H	1.42629182	5.06304947	2.18790205
H	-1.16660342	5.38157686	-1.30973601
H	-1.42629182	-5.06304947	2.18790205
H	1.16660342	-5.38157686	-1.30973601
H	0.16590971	-4.65290280	1.50980795
H	-0.95401923	-5.67518793	0.58539192
H	-0.16590971	4.65290280	1.50980795
H	0.95401923	5.67518793	0.58539192

Table S16. Atom numbers and coordinates (in Å) of stable isohypericin **IIb**, C_i symmetry 'double-butterfly'.

6	0.050484	2.533166	-3.497682
8	-0.586055	3.400698	-4.274109
6	1.293065	2.041944	-3.935691
6	2.043378	1.165180	-3.176057
6	3.210899	0.488702	-3.865886
6	1.598367	0.816816	-1.854811
6	2.392907	0.058017	-0.910103
6	3.823833	0.119893	-0.867234
8	4.511413	1.077398	-1.521576

6	4.569310	-0.756999	-0.104830
6	3.925110	-1.647331	0.764170
8	4.673183	-2.486612	1.465345
6	2.522833	-1.555945	0.936371
6	1.764155	-0.674000	0.117549
6	1.895172	-2.296439	2.002402
8	2.541672	-3.145269	2.679275
6	0.506473	-2.003271	2.318435
6	-0.258994	-1.121390	1.508373
6	-0.050484	-2.533166	3.497682
8	0.586055	-3.400698	4.274109
6	-1.293065	-2.041944	3.935691
6	-2.043378	-1.165180	3.176057
6	-1.598367	-0.816816	1.854811
6	-2.392907	-0.058017	0.910103
6	-3.823833	-0.119893	0.867234
6	-4.569310	0.756999	0.104830
6	-3.925110	1.647331	-0.764170
8	-4.673183	2.486612	-1.465345
6	-2.522833	1.555945	-0.936371
6	-1.764155	0.674000	-0.117549
6	-1.895172	2.296439	-2.002402
6	-0.506473	2.003271	-2.318435
6	0.258994	1.121390	-1.508373
6	-0.357558	0.469617	-0.375923
6	0.357558	-0.469617	0.375923
8	-4.511413	-1.077398	1.521576
6	-3.210899	-0.488702	3.865886
1	1.612557	2.319147	-4.941378
1	5.656187	-0.701937	-0.115228
1	-1.612557	-2.319147	4.941378
1	-5.656187	0.701937	0.115228

1	4.055454	-2.970578	2.075721
1	1.472823	-3.553348	3.851283
1	-1.472823	3.553348	-3.851283
1	-4.055454	2.970578	-2.075721
1	-2.905197	-0.244766	4.891798
1	-3.888733	-1.743778	1.848357
1	2.905197	0.244766	-4.891798
1	3.888733	1.743778	-1.848357
8	-2.541672	3.145269	-2.679275
1	3.498625	-0.441361	-3.361815
1	4.105336	1.123136	-3.929437
1	-4.105336	-1.123136	3.929437
1	-3.498625	0.441361	3.361815

Table S17. Atom numbers and coordinates (in Å) of stable isohypericin **IIb**, C_2 symmetry 'propeller'.

6	-2.236841	3.660218	0.412345
8	-2.236841	4.986785	0.456513
6	-3.431079	2.985812	0.725174
6	-3.542500	1.610530	0.640199
6	-4.788438	0.983761	1.232045
6	-2.430776	0.840831	0.155053
6	-2.488269	-0.577325	-0.144480
6	-3.666919	-1.232863	-0.626929
8	-4.733665	-0.535710	-1.067997
6	-3.747842	-2.608040	-0.728394
6	-2.622254	-3.398529	-0.459281
8	-2.751538	-4.715864	-0.527880
6	-1.379851	-2.771657	-0.203273
6	-1.313463	-1.354599	-0.100939
6	-0.193714	-3.576674	-0.035290
8	-0.252276	-4.838268	-0.050893

6	1.081004	-2.900465	0.154717
6	1.172238	-1.484777	0.078208
6	2.236841	-3.660218	0.412345
8	2.236841	-4.986785	0.456513
6	3.431079	-2.985812	0.725174
6	3.542500	-1.610530	0.640199
6	2.430776	-0.840831	0.155053
6	2.488269	0.577325	-0.144480
6	3.666919	1.232863	-0.626929
6	3.747842	2.608040	-0.728394
6	2.622254	3.398529	-0.459281
8	2.751538	4.715864	-0.527880
6	1.379851	2.771657	-0.203273
6	1.313463	1.354599	-0.100939
6	0.193714	3.576674	-0.035290
8	0.252276	4.838268	-0.050893
6	-1.081004	2.900465	0.154717
6	-1.172238	1.484777	0.078208
6	0.032865	0.697679	-0.008991
6	-0.032865	-0.697679	-0.008991
8	4.733665	0.535710	-1.067997
6	4.788438	-0.983761	1.232045
1	-4.262239	3.587421	1.096051
1	-4.666037	-3.074960	-1.079588
1	4.262239	-3.587421	1.096051
1	4.666037	3.074960	-1.079588
1	-1.849879	-5.096083	-0.356310
1	1.304721	-5.271321	0.263438
1	-1.304721	5.271321	0.263438
1	1.849879	5.096083	-0.356310
1	5.040792	-1.526913	2.152234

1	4.487427	-0.397882	-1.146971
1	-5.040792	1.526913	2.152234
1	-4.487427	0.397882	-1.146971
1	-4.631624	-0.071246	1.485879
1	-5.660634	1.039411	0.566335
1	4.631624	0.071246	1.485879
1	5.660634	-1.039411	0.566335

Table S18. Atom numbers and coordinates (in Å) of stable isohypericin **IIc**, C_1 symmetry 'double-butterfly'.

6	-3.615895	2.363411	0.066962
8	-4.936878	2.404227	0.186778
6	-2.947852	3.546684	-0.293597
6	-1.574575	3.609876	-0.406469
6	-1.014000	4.846472	-1.069416
6	-0.784674	2.463730	-0.050765
6	0.659966	2.475176	0.049813
6	1.408290	3.633302	0.436768
8	0.720583	4.674082	0.947618
6	2.787066	3.674465	0.361502
6	3.509205	2.520934	0.026090
8	4.828131	2.612383	-0.055728
6	2.817491	1.299331	-0.126503
6	1.393313	1.275895	-0.074886
6	3.577806	0.080171	-0.246435
8	4.835506	0.105425	-0.377654
6	2.868605	-1.183442	-0.158178
6	1.448894	-1.221064	-0.098412
6	3.610026	-2.374582	-0.034360
8	4.933447	-2.418169	-0.130445
6	2.933483	-3.555352	0.314387

6	1.555242	-3.609875	0.402131
6	0.776935	-2.461273	0.029911
6	-0.667555	-2.471076	-0.085452
6	-1.409811	-3.628313	-0.486092
6	-2.785713	-3.681461	-0.388335
6	-3.503100	-2.536217	-0.017704
8	-4.820789	-2.631327	0.088815
6	-2.817176	-1.307709	0.132801
6	-1.397770	-1.274289	0.061875
6	-3.576676	-0.088608	0.272725
6	-2.870676	1.175014	0.174843
6	-1.450399	1.222016	0.088403
6	-0.696298	-0.009822	0.070961
6	0.694478	0.012473	-0.085265
8	-0.806002	-4.702316	-1.036191
6	0.961448	-4.839969	1.058138
1	-3.565827	4.406433	-0.556719
1	3.343118	4.575575	0.624223
1	3.542532	-4.416802	0.592136
1	-3.319404	-4.585654	-0.674933
1	5.158445	1.688129	-0.230130
1	5.232362	-1.483336	-0.284210
1	-5.230000	1.467823	0.346217
1	-5.152157	-1.712182	0.276478
1	1.604936	-5.119235	1.902799
1	0.112730	-4.473750	-1.241449
1	-1.665818	5.108903	-1.913830
1	1.345763	5.365116	1.207273
8	-4.831092	-0.117219	0.428043
1	-0.001533	4.684253	-1.456951
1	-0.984874	5.698787	-0.380028
1	0.903964	-5.706226	0.385263

1	-0.047951	-4.649925	1.441336

Table S19. Atom numbers and coordinates (in Å) of stable isohypericin **IIc**, C_1 symmetry 'propeller'.

6	-3.589873	-2.350576	0.414040
8	-4.916823	-2.391508	0.450855
6	-2.879995	-3.520651	0.734646
6	-1.500947	-3.587200	0.652774
6	-0.834893	-4.810401	1.248819
6	-0.766053	-2.454335	0.164655
6	0.652703	-2.470081	-0.137444
6	1.339154	-3.629433	-0.621249
8	0.670846	-4.719050	-1.054198
6	2.715205	-3.669201	-0.732667
6	3.473382	-2.520050	-0.469913
8	4.793797	-2.609310	-0.548946
6	2.811403	-1.297699	-0.209130
6	1.394762	-1.272438	-0.096510
6	3.581952	-0.086340	-0.048366
8	4.845306	-0.111436	-0.075100
6	2.871369	1.164666	0.148353
6	1.450529	1.218075	0.087308
6	3.599628	2.341321	0.397423
8	4.926105	2.379555	0.428567
6	2.894229	3.515680	0.716024
6	1.517965	3.591193	0.644227
6	0.773544	2.458165	0.169984
6	-0.647000	2.473352	-0.119757
6	-1.344743	3.630094	-0.594289
6	-2.721116	3.657229	-0.719641
6	-3.477642	2.502209	-0.475648

8	-4.797052	2.587859	-0.563435
6	-2.808648	1.288371	-0.210570
6	-1.388473	1.273408	-0.094153
6	-3.578056	0.078242	-0.046806
8	-4.841690	0.100039	-0.076126
6	-2.865398	-1.171898	0.155080
6	-1.447131	-1.216186	0.084048
6	-0.696093	0.012906	-0.001506
6	0.700745	-0.010147	-0.000404
8	-0.601397	4.683641	-0.987801
6	0.880394	4.825593	1.236905
1	-3.455303	-4.369905	1.106093
1	3.207116	-4.573397	-1.086125
1	3.476803	4.364726	1.076553
1	-3.241019	4.555856	-1.054877
1	5.145631	-1.694644	-0.380888
1	5.234801	1.456055	0.231708
1	-5.229546	-1.471300	0.249038
1	-5.145403	1.670455	-0.392518
1	1.419683	5.084780	2.158142
1	-1.189373	5.373728	-1.325234
1	-1.368683	-5.075826	2.170889
1	-0.269572	-4.499383	-1.129900
1	0.215116	-4.619395	1.499873
1	-0.863912	-5.686321	0.586320
1	-0.173514	4.662873	1.490867
1	0.939032	5.680165	0.552108

Table S20. Atom numbers and coordinates (in Å) of stable fringelite D **IIIa**, C_{2h} symmetry 'double-butterfly'.

6	0.016226	3.564640	-2.448805
8	0.110023	4.885626	-2.510607
6	-0.304779	2.870590	-3.623304
6	-0.406750	1.492574	-3.617517
8	-0.934285	0.842297	-4.669623
6	-0.044126	0.720755	-2.465075
6	0.044126	-0.720755	-2.465075
6	0.406750	-1.492574	-3.617517
8	0.934285	-0.842297	-4.669623
6	0.304779	-2.870590	-3.623304
6	-0.016226	-3.564640	-2.448805
8	-0.110023	-4.885626	-2.510607
6	-0.139806	-2.848301	-1.239096
6	-0.075854	-1.425004	-1.248083
6	-0.239872	-3.581477	-0.000000
8	-0.368858	-4.843480	-0.000000
6	-0.139806	-2.848301	1.239096
6	-0.075854	-1.425004	1.248083
6	-0.016226	-3.564640	2.448805
8	-0.110023	-4.885626	2.510607
6	0.304779	-2.870590	3.623304
6	0.406750	-1.492574	3.617517
6	0.044126	-0.720755	2.465075
6	-0.044126	0.720755	2.465075
6	-0.406750	1.492574	3.617517
6	-0.304779	2.870590	3.623304
6	0.016226	3.564640	2.448805
8	0.110023	4.885626	2.510607
6	0.139806	2.848301	1.239096
6	0.075854	1.425004	1.248083
6	0.239872	3.581477	0.000000
8	0.368858	4.843480	0.000000

6	0.139806	2.848301	-1.239096
6	0.075854	1.425004	-1.248083
6	0.074153	0.696402	0.000000
6	-0.074153	-0.696402	-0.000000
8	-0.934285	0.842297	4.669623
8	0.934285	-0.842297	4.669623
1	-0.546697	3.450925	-4.515001
1	0.546697	-3.450925	-4.515001
1	0.546697	-3.450925	4.515001
1	-0.546697	3.450925	4.515001
1	-0.262252	-5.192811	-1.573488
1	-0.262252	-5.192811	1.573488
1	0.262252	5.192811	-1.573488
1	0.262252	5.192811	1.573488
1	1.144385	-1.487052	5.358692
1	-1.144385	1.487052	5.358692
1	-1.144385	1.487052	-5.358692
1	1.144385	-1.487052	-5.358692

Table S21. Atom numbers and coordinates (in Å) of stable fringelite D **IIIa**, D_2 symmetry 'propeller'.

C	-3.54338591	-2.43103785	-0.41038516
O	-4.86715877	-2.48945926	-0.46382740
C	-2.81816127	-3.60222385	-0.67086293
C	-1.43786699	-3.60829450	-0.59180751
O	-0.73237529	-4.66631757	-1.02897771
C	-0.71017774	-2.46199136	-0.13281654
C	0.71017774	-2.46199136	0.13281654
C	1.43786699	-3.60829450	0.59180751
O	0.73237529	-4.66631757	1.02897771
C	2.81816127	-3.60222385	0.67086293
C	3.54338591	-2.43103785	0.41038516

O	4.86715877	-2.48945926	0.46382740
C	2.84478289	-1.22902323	0.17037978
C	1.42300567	-1.24496470	0.08607615
C	3.58378010	-0.00000000	0.00000000
O	4.85175774	-0.00000000	0.00000000
C	2.84478289	1.22902323	-0.17037978
C	1.42300567	1.24496470	-0.08607615
C	3.54338591	2.43103785	-0.41038516
O	4.86715877	2.48945926	-0.46382740
C	2.81816127	3.60222385	-0.67086293
C	1.43786699	3.60829450	-0.59180751
C	0.71017774	2.46199136	-0.13281654
C	-0.71017774	2.46199136	0.13281654
C	-1.43786699	3.60829450	0.59180751
C	-2.81816127	3.60222385	0.67086293
C	-3.54338591	2.43103785	0.41038516
O	-4.86715877	2.48945926	0.46382740
C	-2.84478289	1.22902323	0.17037978
C	-1.42300567	1.24496470	0.08607615
C	-3.58378010	-0.00000000	0.00000000
O	-4.85175774	-0.00000000	0.00000000
C	-2.84478289	-1.22902323	-0.17037978
C	-1.42300567	-1.24496470	-0.08607615
C	-0.69911945	0.00000000	0.00000000
C	0.69911945	0.00000000	0.00000000
O	-0.73237529	4.66631757	1.02897771
O	0.73237529	4.66631757	-1.02897771
H	-3.36778989	-4.48872057	-0.99104303
H	3.36778989	-4.48872057	0.99104303
H	3.36778989	4.48872057	-0.99104303
H	-3.36778989	4.48872057	0.99104303

H	5.18948388	-1.56053521	0.29056537
H	5.18948388	1.56053521	-0.29056537
H	-5.18948388	-1.56053521	-0.29056537
H	-5.18948388	1.56053521	0.29056537
H	1.34982586	5.35136239	-1.32024155
H	-1.34982586	5.35136239	1.32024155
H	-1.34982586	-5.35136239	-1.32024155
H	1.34982586	-5.35136239	1.32024155

Table S22. Atom numbers and coordinates (in Å) of stable fringelite D **IIIb**, C_{2h} symmetry 'double-butterfly'.

6	-1.653988	3.795008	1.268515
8	-2.628190	4.685570	1.162301
6	-0.542864	4.126401	2.052084
6	0.489074	3.224432	2.213653
8	1.574372	3.707682	2.860046
6	0.410697	1.884157	1.705881
6	1.373664	0.843012	2.007766
6	2.125713	0.771936	3.228292
8	1.867077	1.552132	4.302363
6	3.160350	-0.125909	3.395478
6	3.424147	-1.089546	2.415574
8	4.441612	-1.913988	2.611826
6	2.550280	-1.202504	1.309245
6	1.506719	-0.255179	1.125008
6	2.685940	-2.339627	0.427577
8	3.662318	-3.135503	0.542901
6	1.653696	-2.572818	-0.556743
6	0.604692	-1.633812	-0.752308
6	1.653988	-3.795008	-1.268515

8	2.628190	-4.685570	-1.162301
6	0.542864	-4.126401	-2.052084
6	-0.489074	-3.224432	-2.213653
6	-0.410697	-1.884157	-1.705881
6	-1.373664	-0.843012	-2.007766
6	-2.125713	-0.771936	-3.228292
6	-3.160350	0.125909	-3.395478
6	-3.424147	1.089546	-2.415574
8	-4.441612	1.913988	-2.611826
6	-2.550280	1.202504	-1.309245
6	-1.506719	0.255179	-1.125008
6	-2.685940	2.339627	-0.427577
8	-3.662318	3.135503	-0.542901
6	-1.653696	2.572818	0.556743
6	-0.604692	1.633812	0.752308
6	-0.537356	0.443906	-0.067796
6	0.537356	-0.443906	0.067796
8	-1.867077	-1.552132	-4.302363
8	-1.574372	-3.707682	-2.860046
1	-0.457871	5.125300	2.476019
1	3.725900	-0.130536	4.325465
1	0.457871	-5.125300	-2.476019
1	-3.725900	0.130536	-4.325465
1	4.421837	-2.566204	1.859819
1	3.281837	-4.308547	-0.512770
1	-3.281837	4.308547	0.512770
1	-4.421837	2.566204	-1.859819
1	-2.323840	-3.101954	-2.774426
1	-1.025138	-2.016531	-4.195532
1	2.323840	3.101954	2.774426
1	1.025138	2.016531	4.195532

Table S23. Atom numbers and coordinates (in Å) of stable fringelite D **IIIb**, D_2 symmetry 'propeller'.

6	-3.615027	-2.339188	0.361220
8	-4.935306	-2.355124	0.465808
6	-2.951529	-3.560875	0.196164
6	-1.573067	-3.604819	0.129848
8	-1.051466	-4.821575	-0.148757
6	-0.767432	-2.432962	0.319238
6	0.679354	-2.441157	0.435202
6	1.444160	-3.528634	0.974302
8	0.881481	-4.585086	1.604765
6	2.822932	-3.555454	0.905335
6	3.528212	-2.464994	0.382394
8	4.846515	-2.558370	0.294726
6	2.821124	-1.291092	0.036142
6	1.402490	-1.268069	0.115480
6	3.555955	-0.127808	-0.410567
8	4.811265	-0.172926	-0.555503
6	2.823054	1.088229	-0.687813
6	1.425623	1.166421	-0.442011
6	3.514126	2.211878	-1.195495
8	4.821000	2.210900	-1.410927
6	2.787417	3.353807	-1.553100
6	1.430790	3.425302	-1.306241
6	0.733676	2.390370	-0.598349
6	-0.645597	2.483749	-0.156091
6	-1.301883	3.708150	0.202091
6	-2.658820	3.762522	0.451600
6	-3.427311	2.592304	0.451880
8	-4.732209	2.702594	0.650393
6	-2.779100	1.344115	0.311323

6	-1.381143	1.295003	0.061020
6	-3.555955	0.127808	0.410567
8	-4.811265	0.172926	0.555503
6	-2.865078	-1.141252	0.340348
6	-1.446969	-1.193355	0.265511
6	-0.694037	0.024945	0.080133
6	0.694037	-0.024945	-0.080133
8	-0.645704	4.882574	0.344710
8	0.815689	4.524086	-1.800718
1	-3.525972	-4.476485	0.068052
1	3.365402	-4.409198	1.307316
1	3.283249	4.170232	-2.074962
1	-3.122679	4.715450	0.699593
1	5.165651	-1.684176	-0.057288
1	5.149116	1.313443	-1.133643
1	-5.221470	-1.404733	0.535404
1	-5.093298	1.775467	0.655527
1	-0.146018	4.422143	-1.772073
1	0.311637	4.742874	0.353468
1	-0.113815	-4.749984	-0.376308
1	-0.051804	-4.415033	1.794913

Table S24. Atom numbers and coordinates (in Å) of stable fringelite D **IIIc**, C_2 symmetry 'propeller'.

6	-3.287928	-2.772924	-0.466910
8	-4.598045	-2.951041	-0.556059
6	-2.457295	-3.870733	-0.691933
6	-1.085796	-3.773904	-0.549690
8	-0.410659	-4.863520	-0.947088
6	-0.459867	-2.545971	-0.114258
6	0.958644	-2.382063	0.154832
6	1.834381	-3.425029	0.585455

8	1.276911	-4.622487	0.941947
6	3.196819	-3.271277	0.702384
6	3.788689	-2.026958	0.448025
8	5.108023	-1.939396	0.509335
6	2.951612	-0.924854	0.189470
6	1.540597	-1.090743	0.103512
6	3.558423	0.374438	-0.010918
8	4.820065	0.492958	-0.022856
6	2.702030	1.515764	-0.193303
6	1.285756	1.396476	-0.081473
6	3.287928	2.772924	-0.466910
8	4.598045	2.951041	-0.556059
6	2.457295	3.870733	-0.691933
6	1.085796	3.773904	-0.549690
6	0.459867	2.545971	-0.114258
6	-0.958644	2.382063	0.154832
6	-1.834381	3.425029	0.585455
6	-3.196819	3.271277	0.702384
6	-3.788689	2.026958	0.448025
8	-5.108023	1.939396	0.509335
6	-2.951612	0.924854	0.189470
6	-1.540597	1.090743	0.103512
6	-3.558423	-0.374438	-0.010918
8	-4.820065	-0.492958	-0.022856
6	-2.702030	-1.515764	-0.193303
6	-1.285756	-1.396476	-0.081473
6	-0.694669	-0.077355	0.012254
6	0.694669	0.077355	0.012254
8	-1.276911	4.622487	0.941947
8	0.410659	4.863520	-0.947088
1	-2.887740	-4.820815	-1.001709

1	3.836906	-4.100064	1.008711
1	2.887740	4.820815	-1.001709
1	-3.836906	4.100064	1.008711
1	5.324046	-0.982833	0.311148
1	5.013845	2.067455	-0.370983
1	-5.013845	-2.067455	-0.370983
1	-5.324046	0.982833	0.311148
1	-0.389619	4.973511	-0.405773
1	-1.964610	5.199558	1.303861
1	0.389619	-4.973511	-0.405773
1	1.964610	-5.199558	1.303861

Table S25. Atom numbers and coordinates (in Å) of stable fringelite D **IIIc**, C_s symmetry 'double-butterfly'.

6	-3.566859	-2.112345	1.310834
8	-4.878855	-2.264795	1.223162
6	-2.882201	-2.861918	2.273635
6	-1.513793	-2.761489	2.378216
8	-0.893971	-3.389773	3.421623
6	-0.707809	-1.995127	1.480089
6	0.743290	-1.995029	1.471146
6	1.566811	-3.096880	1.914037
8	1.103314	-4.333105	2.168699
6	2.939015	-2.976959	1.998469
6	3.591326	-1.819451	1.570713
8	4.905961	-1.755563	1.714920
6	2.835747	-0.800121	0.948832
6	1.418401	-0.905770	0.862632
6	3.541734	0.293170	0.329564
8	4.793593	0.423753	0.483182
6	2.805226	1.206118	-0.507860

6	1.387453	1.128619	-0.614500
6	3.531278	2.127780	-1.295298
8	4.844701	2.271301	-1.208910
6	2.849921	2.879589	-2.253861
6	1.476965	2.809087	-2.374175
6	0.682047	2.030710	-1.451868
6	-0.768743	2.010337	-1.428204
6	-1.602738	3.085262	-1.867000
6	-2.971220	2.989648	-1.975077
6	-3.626814	1.828738	-1.550713
8	-4.940181	1.766418	-1.703827
6	-2.869560	0.820143	-0.919702
6	-1.450443	0.925644	-0.823333
6	-3.577899	-0.274903	-0.303648
8	-4.828807	-0.406693	-0.458948
6	-2.839052	-1.185291	0.536406
6	-1.419657	-1.098044	0.646029
6	-0.708714	-0.054644	-0.060410
6	0.676855	0.081516	0.098087
8	-1.010710	4.283989	-2.150151
8	0.985300	3.424448	-3.463914
1	-3.460245	-3.486189	2.956505
1	3.520358	-3.820872	2.364316
1	3.408986	3.500090	-2.951298
1	-3.571400	3.820501	-2.348745
1	5.185939	-0.890357	1.305111
1	5.147756	1.619573	-0.517302
1	-5.185094	-1.611539	0.533239
1	-5.223336	0.902279	-1.291997
1	0.121535	3.818694	-3.260014
1	-1.696376	4.943915	-2.327268
1	-1.563722	-3.775996	4.004097

1	0.244715	-4.278435	2.619159

Table S26. Atom numbers and coordinates (in Å) of stable fringelite D **IIIc**, C_i symmetry 'double-butterfly'.

6	-3.583549	2.430532	-0.010494
8	-4.902475	2.484240	-0.099620
6	-2.896405	3.612851	0.290695
6	-1.523177	3.612049	0.360143
8	-0.892770	4.745087	0.797037
6	-0.718596	2.473899	0.044341
6	0.730364	2.489450	-0.037942
6	1.525127	3.651954	-0.367831
8	1.023295	4.795863	-0.859464
6	2.904235	3.622361	-0.299796
6	3.586706	2.443450	-0.002543
8	4.907422	2.492990	0.085143
6	2.851386	1.242159	0.129231
6	1.426332	1.260212	0.070466
6	3.577158	0.006630	0.235350
8	4.837947	-0.004216	0.370862
6	2.845523	-1.236490	0.131988
6	1.422872	-1.247905	0.068050
6	3.583549	-2.430532	0.010494
8	4.902475	-2.484240	0.099620
6	2.896405	-3.612851	-0.290695
6	1.523177	-3.612049	-0.360143
6	0.718596	-2.473899	-0.044341
6	-0.730364	-2.489450	0.037942
6	-1.525127	-3.651954	0.367831
6	-2.904235	-3.622361	0.299796
6	-3.586706	-2.443450	0.002543
8	-4.907422	-2.492990	-0.085143

6	-2.851386	-1.242159	-0.129231
6	-1.426332	-1.260212	-0.070466
6	-3.577158	-0.006630	-0.235350
8	-4.837947	0.004216	-0.370862
6	-2.845523	1.236490	-0.131988
6	-1.422872	1.247905	-0.068050
6	-0.697201	-0.005210	-0.067290
6	0.697201	0.005210	0.067290
8	-1.023295	-4.795863	0.859464
8	0.892770	-4.745087	-0.797037
1	-3.476098	4.511379	0.507477
1	3.462341	4.528633	-0.525568
1	3.476098	-4.511379	-0.507477
1	-3.462341	-4.528633	0.525568
1	5.211789	1.559935	0.247913
1	5.200201	-1.540706	0.255972
1	-5.200201	1.540706	-0.255972
1	-5.211789	-1.559935	-0.247913
1	1.557484	-5.390493	-1.077138
1	-0.175470	-4.998377	0.429256
1	-1.557484	5.390493	1.077138
1	0.175470	4.998377	-0.429256

Table S27. Atom numbers and coordinates (in Å) of stable fringelite D **IIId**, C_2 symmetry 'propeller'.

6	-2.510453	3.341238	-1.074948
8	-2.590067	4.628117	-1.379234
6	-3.664223	2.562946	-1.192005
6	-3.671901	1.231375	-0.825060
8	-4.802551	0.574570	-1.137688
6	-2.500601	0.602834	-0.260723
6	-2.449294	-0.756706	0.248913

6	-3.559925	-1.466239	0.800507
8	-4.713206	-0.769892	1.028125
6	-3.511343	-2.795886	1.155512
6	-2.312701	-3.510097	1.039940
8	-2.325774	-4.801297	1.333733
6	-1.146443	-2.812988	0.665920
6	-1.205772	-1.428439	0.331524
6	0.102961	-3.534930	0.609756
8	0.139301	-4.782589	0.824970
6	1.310049	-2.804042	0.302987
6	1.287760	-1.386428	0.154025
6	2.517437	-3.519031	0.172546
8	2.606336	-4.831156	0.327812
6	3.674862	-2.818171	-0.187118
6	3.644747	-1.445907	-0.298178
6	2.490460	-0.656645	-0.005118
6	2.459868	0.795651	0.019492
6	3.588209	1.641980	0.329422
6	3.501701	3.016871	0.229518
6	2.306979	3.644542	-0.130060
8	2.310756	4.961354	-0.274896
6	1.142773	2.860511	-0.283900
6	1.211173	1.445686	-0.146007
6	-0.102371	3.514663	-0.606260
8	-0.138728	4.762890	-0.821572
6	-1.305346	2.721071	-0.678892
6	-1.292318	1.340245	-0.334550
6	-0.019850	0.681492	-0.117554
6	0.020267	-0.695828	0.120026
8	4.751075	1.192741	0.832836
8	4.758988	-0.801921	-0.756996

1	-4.574685	3.002829	-1.594098
1	-4.389414	-3.310590	1.548257
1	4.584410	-3.384146	-0.393453
1	4.381880	3.616691	0.452267
1	-1.389912	-5.121303	1.206001
1	1.688858	-5.142299	0.564413
1	-1.677697	5.002178	-1.236034
1	1.380141	5.213694	-0.526147
1	5.425806	-1.456431	-1.010003
1	4.979799	0.340982	0.425339
1	-4.986727	-0.103126	-0.466368
1	-5.344398	-1.338531	1.492118

Table S28. Atom numbers and coordinates (in Å) of stable fringelite D **IIId**, C_s symmetry 'double-butterfly'.

6	-3.566859	-2.112345	1.310834
8	-4.878855	-2.264795	1.223162
6	-2.882201	-2.861918	2.273635
6	-1.513793	-2.761489	2.378216
8	-0.893971	-3.389773	3.421623
6	-0.707809	-1.995127	1.480089
6	0.743290	-1.995029	1.471146
6	1.566811	-3.096880	1.914037
8	1.103314	-4.333105	2.168699
6	2.939015	-2.976959	1.998469
6	3.591326	-1.819451	1.570713
8	4.905961	-1.755563	1.714920
6	2.835747	-0.800121	0.948832
6	1.418401	-0.905770	0.862632
6	3.541734	0.293170	0.329564

8	4.793593	0.423753	0.483182
6	2.805226	1.206118	-0.507860
6	1.387453	1.128619	-0.614500
6	3.531278	2.127780	-1.295298
8	4.844701	2.271301	-1.208910
6	2.849921	2.879589	-2.253861
6	1.476965	2.809087	-2.374175
6	0.682047	2.030710	-1.451868
6	-0.768743	2.010337	-1.428204
6	-1.602738	3.085262	-1.867000
6	-2.971220	2.989648	-1.975077
6	-3.626814	1.828738	-1.550713
8	-4.940181	1.766418	-1.703827
6	-2.869560	0.820143	-0.919702
6	-1.450443	0.925644	-0.823333
6	-3.577899	-0.274903	-0.303648
8	-4.828807	-0.406693	-0.458948
6	-2.839052	-1.185291	0.536406
6	-1.419657	-1.098044	0.646029
6	-0.708714	-0.054644	-0.060410
6	0.676855	0.081516	0.098087
8	-1.010710	4.283989	-2.150151
8	0.985300	3.424448	-3.463914
1	-3.460245	-3.486189	2.956505
1	3.520358	-3.820872	2.364316
1	3.408986	3.500090	-2.951298
1	-3.571400	3.820501	-2.348745
1	5.185939	-0.890357	1.305111
1	5.147756	1.619573	-0.517302
1	-5.185094	-1.611539	0.533239
1	-5.223336	0.902279	-1.291997

1	0.121535	3.818694	-3.260014
1	-1.696376	4.943915	-2.327268
1	-1.563722	-3.775996	4.004097
1	0.244715	-4.278435	2.619159

References

S1. Salthouse, J. A.; Ware, M. J. Point group character tables and related data. Cambridge University Press, Cambridge 1972.
